# Compartmentalization of the proteasome-interacting proteins during sperm capacitation

**DOI:** 10.1038/s41598-019-49024-0

**Published:** 2019-08-29

**Authors:** Michal Zigo, Pavla Manaskova-Postlerova, Vera Jonakova, Karl Kerns, Peter Sutovsky

**Affiliations:** 1 Division of Animal Sciences, University of Missouri, Columbia, MO 65211 USA; 2 Laboratory of Reproductive Biology, Institute of Biotechnology of the Czech Academy of Sciences, Vestec, 25250 Czech Republic; 3 Department of Veterinary Sciences, Faculty of Agrobiology, Food and Natural Resources, Czech University of Life Sciences, Prague, Czech Republic; 4 Department of Obstetrics, Gynecology & Women’s Health, University of Missouri, Columbia, MO 65211 USA

**Keywords:** Proteomics, Reproductive biology

## Abstract

Ubiquitination is a stable, reversible posttranslational modification of target proteins by covalent ligation of the small chaperone protein ubiquitin. Most commonly ubiquitination targets proteins for degradation/recycling by the 26S proteasome in a well-characterized enzymatic cascade. Studies using human and non-human mammalian spermatozoa revealed the role of the ubiquitin-proteasome system (UPS) in the regulation of fertilization, including sperm-zona pellucida (ZP) interactions as well as the early events of sperm capacitation, the remodeling of the sperm plasma membrane and acrosome, and for the acquisition of sperm fertilizing ability. The present study investigated the activity of UPS during *in vitro* capacitation of fresh boar spermatozoa in relation to changes in sperm proteome. Parallel and sequential treatments of ejaculated and capacitated spermatozoa under proteasome permissive/inhibiting conditions were used to isolate putative sperm proteasome-associated sperm proteins in a compartment-specific manner. A differential proteomic approach employing 1D PAGE revealed differences in accumulated proteins at the molecular weights of 60, 58, 49, and 35 kDa, and MS analysis revealed the accumulation of proteins previously reported as proteasome co-purifying proteins, as well as some novel proteins. Among others, P47/lactadherin, ACRBP, ADAM5, and SPINK2 (alias SAAI) were processed by the proteasome in a capacitation dependent manner. Furthermore, the capacitation-induced reorganization of the outer acrosomal membrane was slowed down in the presence of proteasomal inhibitors. These novel results support the proposed role of UPS in sperm capacitation and open several new lines of inquiry into sperm capacitation mechanism.

## Introduction

Protein degradation is a crucial part of protein turnover in all living organisms. Sperm-intrinsic extracellular and intracellular proteins are degraded by two distinct mechanisms. In most cell types, extracellular proteins and their cell surface receptors are typically degraded by endocytosis and autophagy, thought to be less selective, while many intracellular proteins are degraded in a substrate-specific, tightly regulated manner by the ubiquitin-proteasome system (UPS). Frequently, the autophagic pathway and UPS intersect^1^.

Degradation of proteins via UPS can be differentiated into two discrete and successive steps, including substrate ubiquitination (covalent tagging with isopeptide chains of 8.5 kDa ubiquitin) and substrate degradation by the 26S proteasome, with concomitant release and disassembly of the polyubiquitin chain. The first step, which is highly evolutionarily conserved, requires the participation of three enzymes, the ubiquitin-activating enzyme (E1/UBE1), the ubiquitin-conjugating enzyme (E2/UBE2), and a ubiquitin ligase (E3/UBE3). The canonical 26S proteasome is composed of a hollow 20S core capped on one or both ends with a 19S regulatory particle. The 19S particle recognizes, engages and removes the multi-ubiquitin tail attached to a substrate protein. The 20S core possesses proteolytic activity with specific affinity for ubiquitin-tagged proteins. Proteins, broken into small peptides by the 20S core, are released in the cytoplasm and further disassembled by soluble cytosolic endopeptidases. The polyubiquitin chain is broken down by deubiquitinating enzymes to single, reusable ubiquitin molecules (reviewed in^2,3^).

Initial studies using human and non-human mammalian spermatozoa^4–9^ revealed the role of UPS in the regulation of fertilization, including sperm-zona pellucida (ZP) interactions that require sperm capacitation, which remodels sperm plasma membrane and acrosome in anticipation of fertilization fertilizing ability^2,10–13^. The involvement of UPS in sperm capacitation is a new paradigm in fertilization biology^14^. Capacitation endows spermatozoa with the ability to recognize, bind and penetrate the oocyte vitelline coat, prior to fusing with the oocyte plasma membrane^15,16^. Capacitation begins with the removal of decapacitating factors that trigger calcium and bicarbonate ion influx, increase intracellular pH, and efflux of cholesterol. The pH shift/alkalinization activates soluble sperm adenylate cyclase, increasing intracellular cAMP and thus activating protein kinase A (PKA)^17^. Recent studies show that PKA phosphorylates 26S proteasome and increases its chymotrypsin-like proteolytic activity^18^. Once activated by PKA, the proteasome could directly or indirectly modulate substrate protein phosphorylation at serine and threonine residues through a feedback loop^4^. Cholesterol efflux at capacitation results in increased fluidity of the sperm plasma membrane, allowing protein redistribution and formation of the *ZP-*binding multiprotein complexes^19–21^. Proteasomal subunits and other UPS components have been identified in these complexes^19,20^, suggesting post-translation modifications of multiprotein complex-associated proteins during capacitation^22^. The degradation of A-kinase anchoring proteins (AKAPs) was hindered by proteasomal inhibitors during bull sperm capacitation^23^. Furthermore, the inhibition of E1/UBE1 prevented the capacitation-induced loss of spermadhesin AQN1 and acrosin inhibitor, decreasing sperm ability to fertilize oocytes *in vitro*^24^. Proteasomal inhibition during sperm capacitation also prevented the de-aggregation of spermadhesins and DQH protein, a prerequisite for sperm detachment from the oviductal epithelium-sperm reservoir^12^. Studies using transgenic GFP-PSMA1, green fluorescent proteasome pig^25^ also identified possible targets of posttranslational modification by UPS during the capacitation, such as lactadherin MFGE-8, A Disintegrin And Metalloproteinase 5 (ADAM5), spermadhesin AWN1, acrosin binding protein (ACRBP), and acrosin inhibitor (SPINK2); however, there is no information on how these proteins are being modulated by UPS during capacitation. Additional sperm proteasome/UPS substrates have been identified and the nature of UPS modulation disclosed^14^. As most of the aforementioned substrates of sperm UPS are localized to the acrosome, proteasomal inhibition during sperm capacitation prevented acrosomal exocytosis^26^. Altogether, these data provide an ample basis for the prediction of sperm UPS being responsible for the capacitation-induced, proteasome-mediated release of spermatozoa from oviductal sperm reservoir.

Although a few targets of UPS during mammalian sperm capacitation have been identified, we still lack the complex proteomic overview of the UPS targets during this sperm maturation process. The exhaustive list of these target proteins will help to fully comprehend the involvement of UPS during sperm capacitation. This information will also shed light on the downstream sequence of events that leads to acrosomal exocytosis and hyperactivation that is still poorly understood, mainly beyond the triggering of protein tyrosine phosphorylation.

Based on previous studies, we hypothesize that UPS, proteasomal substrates and proteasome-interacting proteins regulate mammalian sperm capacitation in preparation for fertilization. The objective of this study is, therefore, to further elucidate the role of UPS in sperm capacitation by pursuing two independent objectives: i) to assess the involvement of UPS in the capacitation-induced posttranslational modifications and redistribution of sperm proteins previously reported to co-purify with 26S proteasome, and ii) to identify new target proteins for UPS during capacitation.

## Results

### Proteasome modulation affects boar sperm capacitation

**Figure 1:**
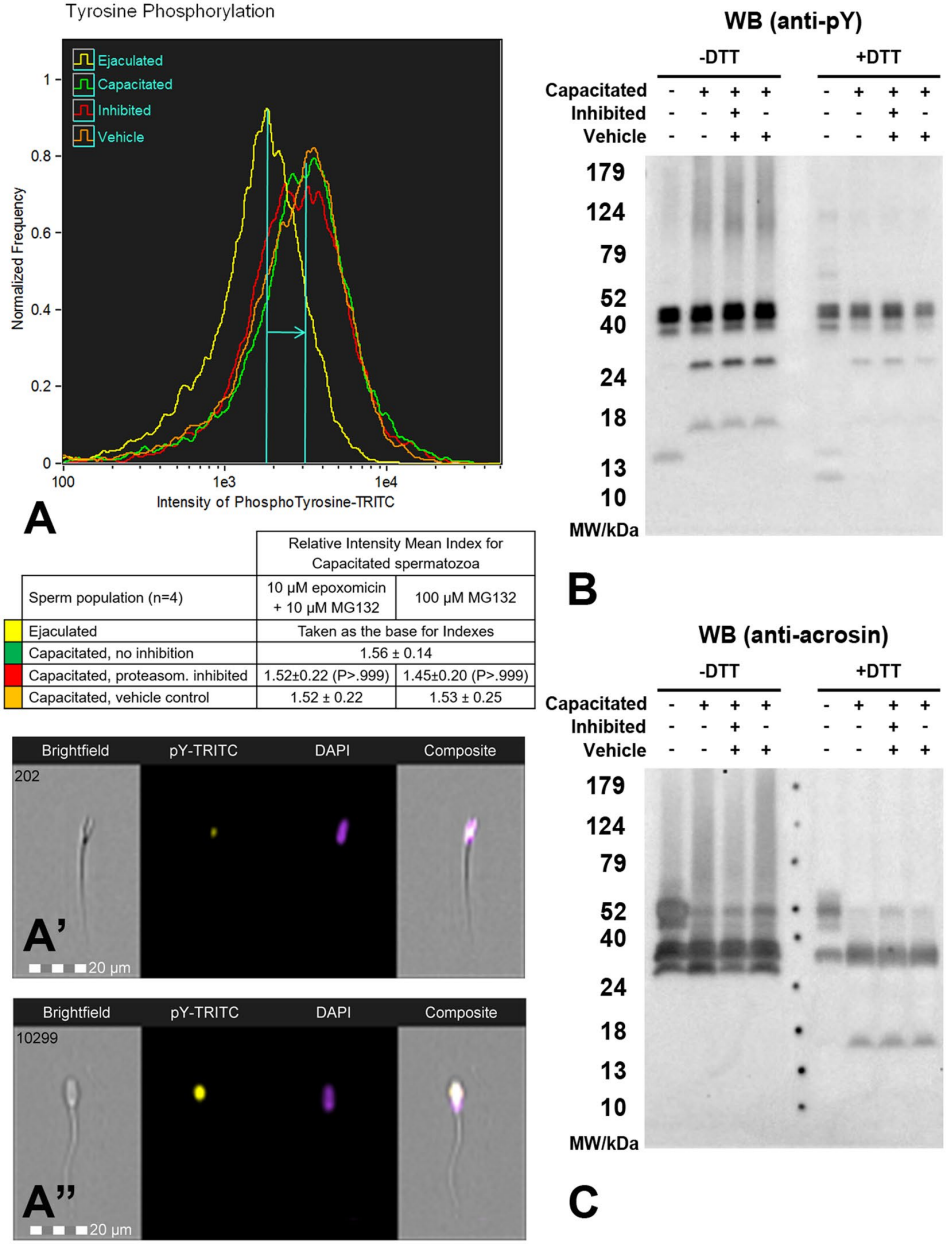
Flow cytometric and Western blot analysis of sperm *in vitro* capacitation. (**A**) In spermatozoa labeled with an anti-phosphotyrosine antibody, increased protein tyrosine phosphorylation is documented by the shift of the mean fluorescence intensity in capacitated spermatozoa (**A**”) compared to ejaculated spermatozoa (**A**’). (**B**) Western blot detection of protein tyrosine phosphorylation in non-reducing and reducing conditions respectively showed unique bands in capacitated spermatozoa regardless of proteasomal inhibition. (**C**) Western blot detection of proacrosin/acrosin conversion in non-reducing and reducing conditions respectively is represented by the decrease of the molar mass of inactive proacrosin (55–53 kDa) in ejaculated spermatozoa to 49, 35 and 25 kDa of active α-, β-, and γ- acrosin, respectively. Proteasomal inhibition at any level neither affected protein tyrosine phosphorylation nor proacrosin/acrosin conversion. Flow cytometry results are presented as mean ± SD of four representative replicates, including the appropriate vehicle and negative controls. Every flow cytometric run represents 10,000 events. Due to the proclivity of target proteins to run differentially, Western blots were performed under both non-reducing and reducing conditions and replicated three times with comparable results. Residual gels after electrotransfer were stained with CBB to show comparable protein loads per well (Fig. S3).

Spermatozoa before *in vitro* capacitation (IVC) and after IVC under proteasome permissive vs. inhibiting conditions were assessed for changes in protein tyrosine phosphorylation (Fig. 1A,B) and proacrosin/acrosin conversion (Fig. 1C), both of which are hallmarks of boar sperm capacitation^27–29^, using FlowSight image-based flow cytometer (IBFC) and Western Blotting. Protein tyrosine phosphorylation was found to be significantly different (P < 0.001) in capacitated spermatozoa compared to ejaculated spermatozoa (Fig. S4). For proteasome inhibition, two different types of conditions were used throughout the study: i) moderate proteasome inhibiting conditions (10 μM epoxomicin + 10 μM MG132 in 0.2% DMSO), and ii) more potent inhibiting conditions: (100 μM MG132 in 0.2% EtOH). During IVC, an increased tyrosine phosphorylation in the post-acrosomal region, typical labeling for the antibody used in formaldehyde-fixed boar spermatozoa, was observed (Fig. 1A’,A”); this event was mirrored by a shift of relative fluorescence intensity mean in the capacitated spermatozoa compared to ejaculated spermatozoa (1.56 ± 0.14 index change), while proteasomal inhibition had no effect (Figs 1A and S4). The same trend was observed by Western blotting where unique bands were present after IVC, and proteasomal inhibition had no detectable effect on the appearance of such bands (Fig. 1B). We used monoclonal antibody acr-2 that recognizes several forms of boar acrosin (55, 53, 45, and 38 kDa)^30^ for monitoring proacrosin/acrosin conversion during boar sperm IVC. Acrosin activation is represented by the conversion of proacrosin (53–55 kDa) in the ejaculated spermatozoa to active α-acrosin, β-acrosin and γ-acrosin (49, 35, 25 kDa, respectively) in capacitated spermatozoa (Fig. 1C). As in the previous experiment, no statistically significant differences were found between capacitated spermatozoa with and without proteasomal inhibition.

### Proteasomal activity contributes to sperm membrane remodeling during capacitation

**Figure 2:**
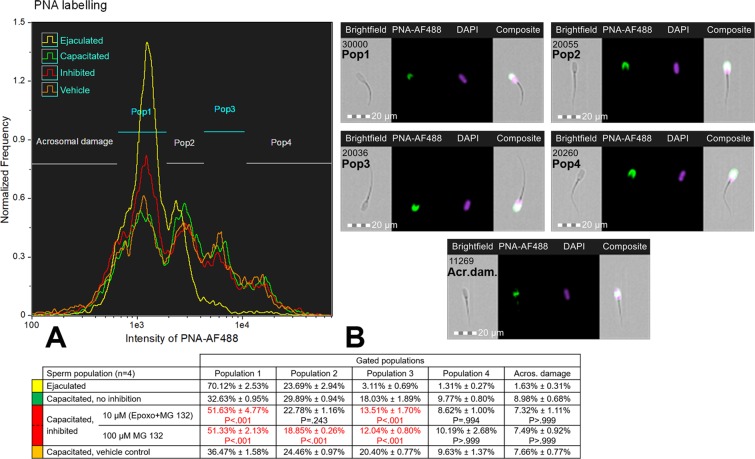
(**A**) Flow cytometric measurements of the outer acrosomal membrane (OAM) remodeling during *in vitro* capacitation under the proteasomal activity permissive (green)/inhibiting (red) conditions with vehicle (orange) and non-capacitated (ejaculated, yellow) control. Fresh, non-fixed spermatozoa were used and labeled with lectin PNA (peanut agglutinin). 0.2% (v/v) DMSO was used as a vehicle for both mild (10 μM epoxomicin + 10 μM MG132) and strong (100 µM MG132) proteasome inhibiting conditions. Four sperm populations with intact acrosomes were distinguished and gated; the representative epifluorescence images of each population are shown in panel B. Population gating and comparisons revealed that populations 1, 2, and 3 differ significantly (P < 0.05, marked red) when compared to vehicle control, depending on proteasomal inhibition during capacitation. Results are presented as mean ± SD of four independent replicates, including the appropriate vehicle and negative controls. Every flow cytometric run represents 10,000 events.

Initially, the flow cytometric sperm screening with lectin PNA (used routinely for acrosomal integrity screening) was performed on fresh, non-fixed, 0.1% TrX-100 permeabilized spermatozoa in order to study the remodeling of the outer acrosomal membrane (OAM) during IVC with and without proteasomal inhibition, using moderate (10 μM epoxomicin + 10 μM MG132 in 0.2% DMSO) or more potent (100 µM MG132 in 0.2% DMSO) proteasome inhibiting conditions (Fig. 2). In ejaculated spermatozoa prior to IVC, three distinct sperm populations were observed (Pop1, Pop2, and Pop3) while a fourth population (Pop4) emerged after IVC (Fig. 2A). Immunofluorescence imaging (Fig. 2B) revealed different intensity and area of PNA labeling in each studied population. In Pop1, low intensity and smaller labeled area limited to the acrosomal ridge were observed; both the intensity and the area of labeling increased and spread towards the post-acrosomal region in order of Pop2 < Pop3 < Pop4 (Figs 2B and S5A). Furthermore, population gating revealed the increased susceptibility of this subpopulation to proteasomal inhibition: 36.47% of non-inhibited post-IVC vehicle control spermatozoa were within Pop1 gate vs. 51.63% (P < 0.001) in moderate and 51.33% (P < 0.001) in more potent proteasome inhibiting conditions and Pop3 had 20.40% in non-inhibited vehicle control group vs. 13.51% (P < 0.001) in moderate and 12.04% (P < 0.001) in more potent proteasome-inhibited groups. Statistical significance (P < 0.001) was also observed in Pop2 between non-inhibited vehicle control and 100 µM MG132 inhibited post-IVC spermatozoa; however, vehicle and the choice of inhibiting conditions had also significant effect (P = 0.04 for vehicle, P = 0.015 for strong vs moderate inhibiting conditions) on this population (see Fig. S6 for bar graph representation of spermatozoa gating with comparisons within each sperm population). The same experiment was replicated with two different boars, but rather with fresh, non-extended spermatozoa for all groups (ejaculated, capacitated with/without proteasomal inhibition, and vehicle control; Fig. S5B,C); which were fixed with formaldehyde and permeabilized with 0.1% TrX-100. Four different populations were detected in IVC spermatozoa as well with significant differences among Pop1 and Pop3 (P < 0.01) depending on proteasomal inhibition. Furthermore, a significant difference (P < 0.05) was observed in ejaculated spermatozoa preincubated in BTS versus non-extended ejaculated spermatozoa, respectively in Pop1 (70.12% vs. 85.99% and 87.04%, respectively) as well as in Pop2 (23.69% vs. 9.95% and 8.73%, respectively). Increased protein tyrosine phosphorylation was observed throughout Pop1 < Pop2 < Pop3 (Fig. S5D).

### Previously reported proteasome co-purifying proteins are affected by proteasomal inhibition during IVC

**Figure 3:**
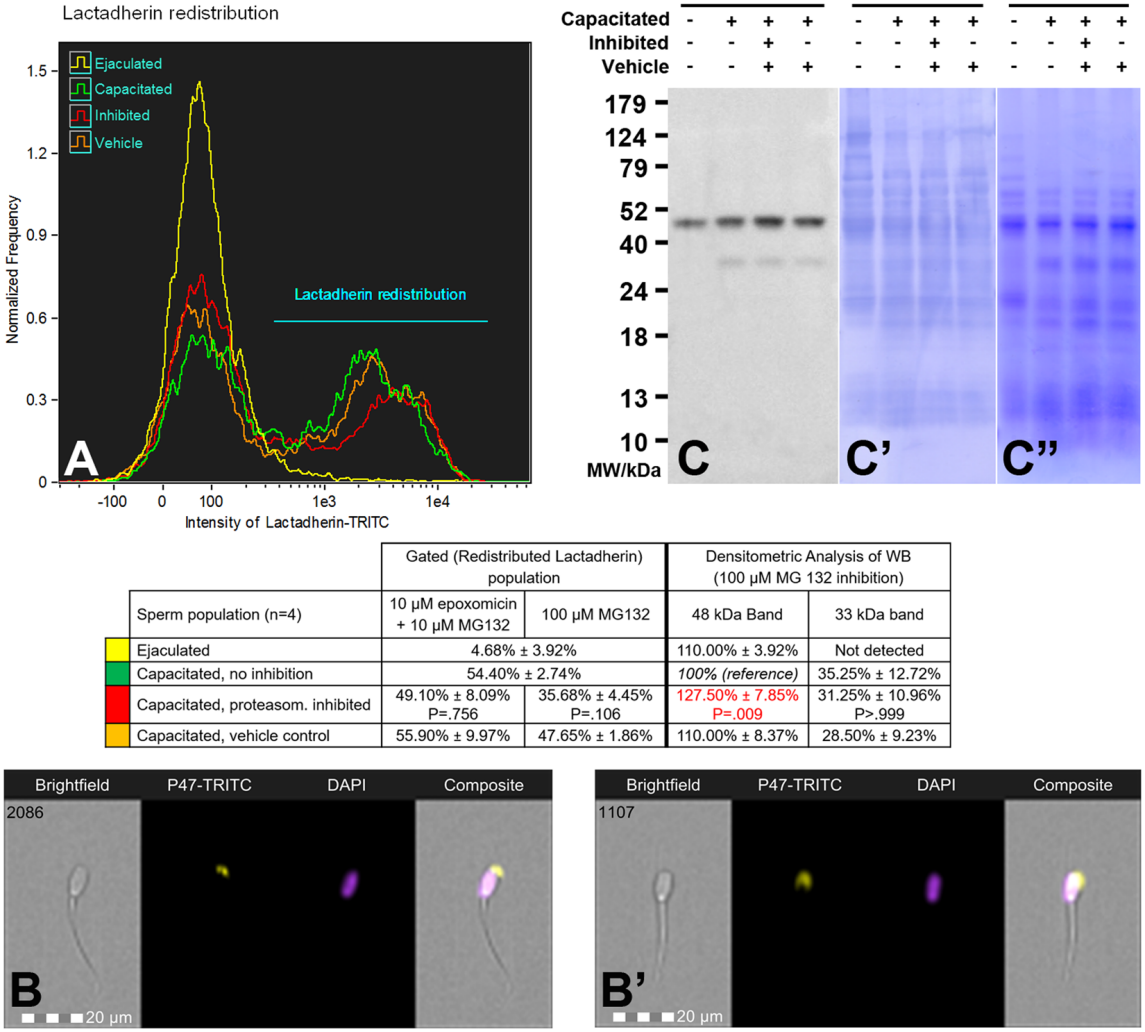
(**A**) Flow cytometric measurements of lactadherin MFGE8 redistribution during *in vitro* capacitation under proteasome permissive/inhibiting conditions (10 µM epoxomicin + 10 µM MG 132 for mild inhibiting conditions, and 100 µM MG132 for strong inhibiting conditions) and vehicle controls, combined with epifluorescence imaging of lactadherin localization in the ejaculated (**B**) and capacitated spermatozoa (**B**’). Every flow cytometric run represents 10,000 events. (**C**) Western blot detection of lactadherin MFGE8 in the ejaculated and capacitated spermatozoa under proteasome permissive/inhibiting conditions (100 μM MG132) including vehicle control, with densitometric analysis; (**C**’) the PVDF membrane stained with CBB after chemiluminescence detection shows comparable protein loads per lane, (**C**”) residual gel after electrotransfer for protein normalization purposes. Proteins were extracted with 1% TrX-100, resolved on a 4–20% gradient gel under reducing conditions, and 20 μg of protein was loaded per single lane. Results are presented as mean ± SD of four independent replicates. Differences in proteasomal inhibition with statistical significance (P < 0.05), when compared to vehicle control, are highlighted in red.

**Figure 4:**
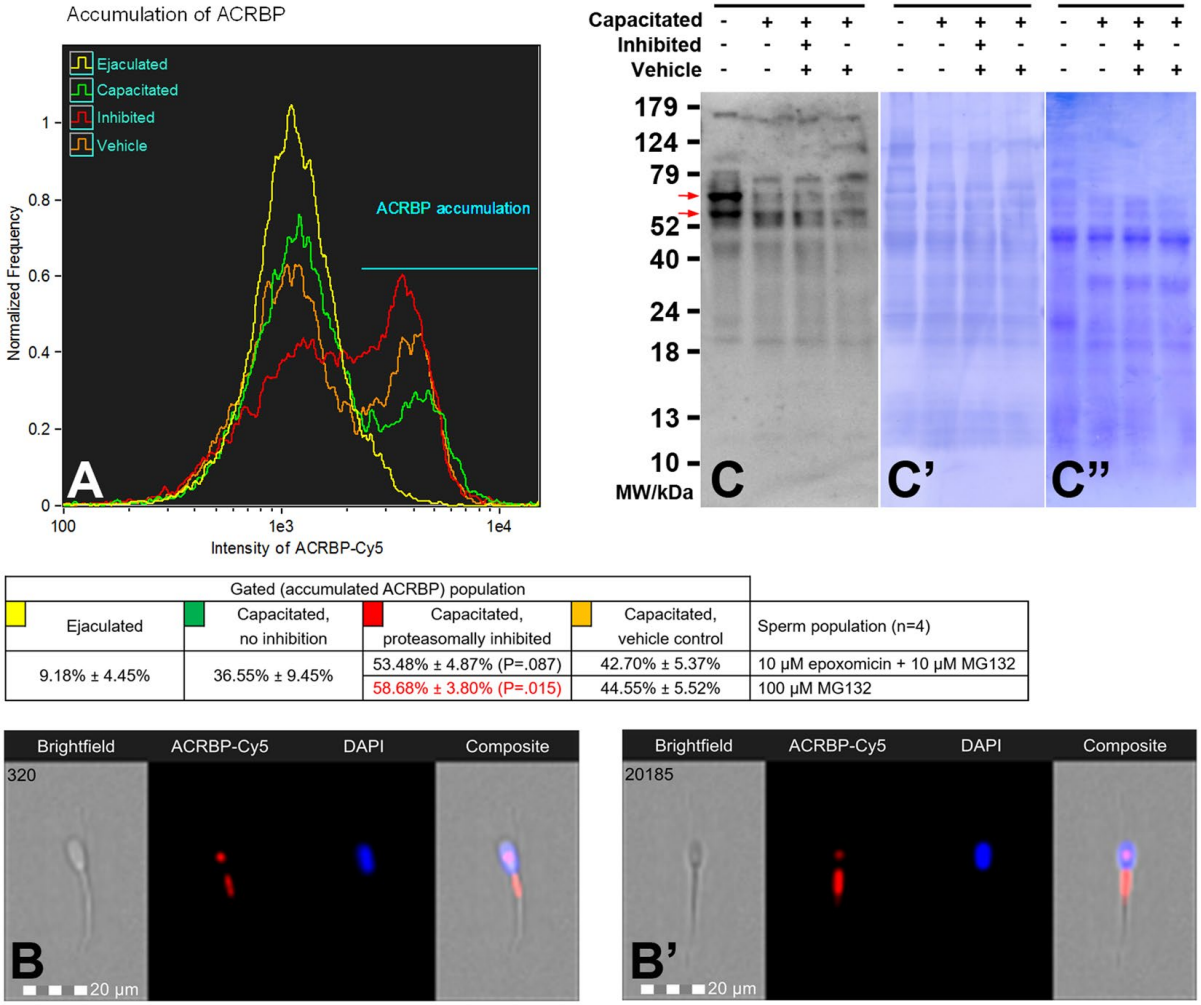
(**A**) Flow cytometric measurements of ACRBP accumulation (immature precursor protein and N-terminal part of the ACRBP degradation products were immunolabeled) during *in vitro* capacitation with proteasome permissive/inhibiting conditions (10 µM epoxomicin +10 µM MG 132 for mild inhibiting conditions, and 100 µM MG132 for strong inhibiting conditions) and vehicle controls, combined with epifluorescence imaging of ACRBP N-terminus in the sperm population with lower fluorescence intensity (**B**), and gated on sperm population with ACRBP accumulated in the midpiece region (**B**’). Every flow cytometric run represents 10,000 events. Differences in proteasomal inhibition with statistical significance (P < 0.05), when compared to vehicle control, are highlighted in red. (**C**) Western blot detection of ACRBP in the ejaculated and capacitated spermatozoa under proteasome permissive/inhibiting conditions (100 μM MG132) including vehicle control, with highlighted (red arrows) 61 and 54 kDa doublet of the immature ACRBP precursor protein; (**C**’) PVDF membrane stained with CBB after chemiluminescence detection shows comparable protein loads per lane, (**C**”) residual gel after electrotransfer for protein normalization purpose. Proteins were extracted with 1% TrX-100, resolved on a 4–20% gradient gel under reducing conditions, and 20 μg of protein was loaded per single lane. Results are presented as mean ± SD of four independent replicates.

**Figure 5:**
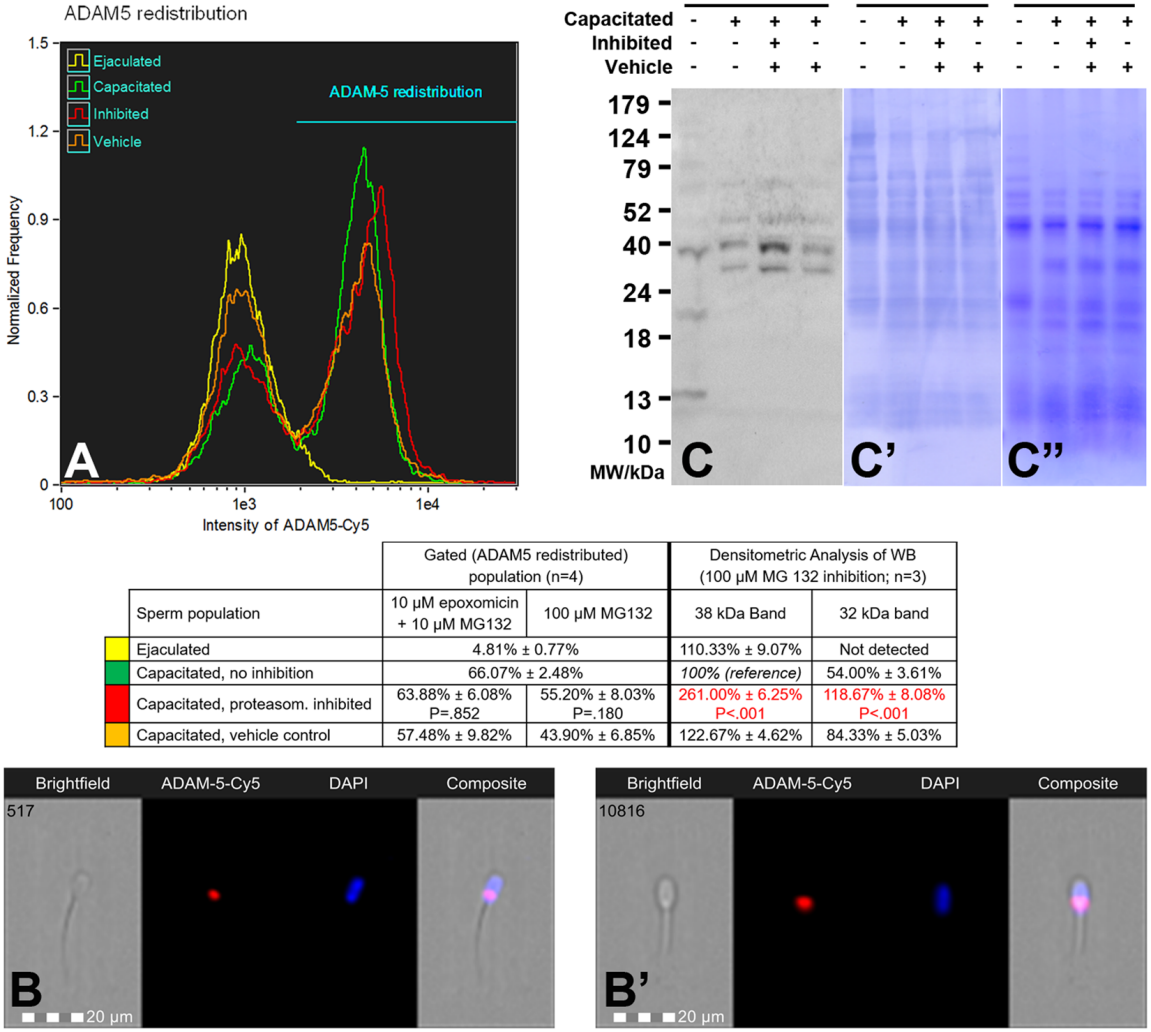
(**A**) Flow cytometric measurements of ADAM5 redistribution/accumulation during *in vitro* capacitation under proteasome permissive/inhibiting conditions (10 µM epoxomicin + 10 µM MG 132 for mild inhibiting conditions, and 100 µM MG132 for strong inhibiting conditions) and vehicle control, combined with epifluorescence imaging of ADAM5 localization in ejaculated (**B**) and capacitated spermatozoa (**B**’). Every flow cytometric run represents 10,000 events. (**C**) Western blot detection of ADAM5 in ejaculated and capacitated spermatozoa under proteasome permissive/inhibiting (100 μM MG132) conditions including vehicle control, with densitometric analysis; (**C**’) PVDF membrane stained with CBB after chemiluminescence detection shows comparable protein loads per lane; (**C**”) residual gel after electrotransfer for protein normalization purposes. Proteins were extracted with 1% TrX-100, resolved on a 4–20% gradient gel under reducing conditions, and 20 μg of protein was loaded per single lane. Results are presented as mean ± SD of four independent replicates for flow cytometry and three independent replicates for Western blotting. Statistically significant differences (P < 0.05), when compared to vehicle control, are highlighted in red.

**Figure 6:**
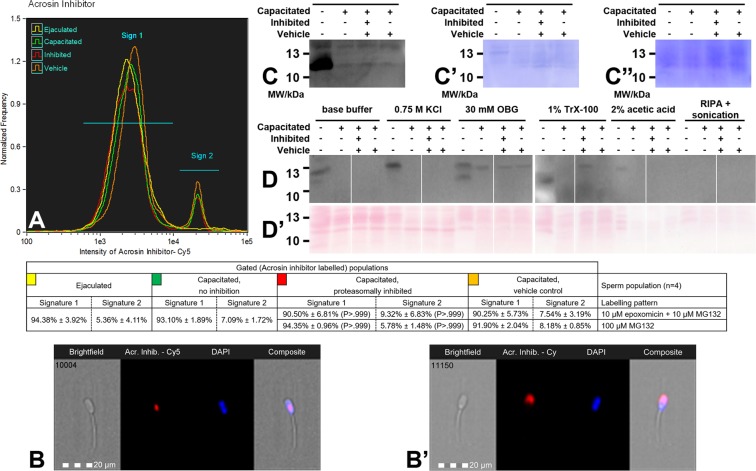
(**A**) Flow cytometric measurements of sperm associated acrosin inhibitor (SPINK2) processing during *in vitro* capacitation under the proteasome permissive/inhibiting conditions (10 µM epoxomicin + 10 µM MG 132 for mild inhibiting conditions, and 100 µM MG132 for strong inhibiting conditions) and vehicle control, combined with epifluorescence imaging of SPINK2 labeling in signature 1 sperm population (**B**) and signature 2 sperm population (**B**’). Every flow cytometric run represents 10,000 events. Differences between treatments were observed, but there was no statistical significance (P > 0.05). (**C**) Western blot detection of SPINK2 extracted with 1% TrX-100 from ejaculated and capacitated sperm under proteasome permissive/inhibiting (100 μM MG132) conditions including vehicle control; (**C**’) PVDF membrane stained with CBB after chemiluminescence detection shows comparable protein loads per lane; (**C**”) residual gel after electrotransfer for protein normalization purposes. Full-length blot at different exposure times is presented in supplemental data as Fig. S16. (**D**) Western blot detection of SPINK2 in sequentially isolated proteins from ejaculated and capacitated sperm with proteasome permissive/inhibiting (10 μM epoxomicin +10 μM MG132 in 0.2% DMSO) conditions and vehicle control; (**D**’) PVDF membrane stained with Ponceau S after electrotransfer for protein normalization purposes. Full-length blot is presented in supplemental data as Fig. S17. For both C and D, proteins were resolved on a 4–20% gradient gel under reducing conditions, and 20 μg of protein was loaded per single lane. Results are presented as mean ± SD of four independent replicates.

Previously reported proteasome co-purifying proteins^25^ were screened after IVC under proteasome permissive and inhibiting conditions by FlowSight cytometer and Western blotting (WB; Figs 3–6).

Flow cytometric histogram of MFGE8 (alias SED1/P47/lactadherin; Fig. 4A) revealed two distinct sperm populations. In the population with lower fluorescence intensity where the majority of ejaculated spermatozoa were present, MFGE8 was localized in the apical ridge of the acrosomal region (Fig. 3B), while the capacitated spermatozoa were predominantly found in the population with higher fluorescence intensity, wherein MFGE8 was redistributed throughout the entire acrosomal region (Fig. 3B’). This redistribution was hindered (P = 0.106, Fig. S7) in spermatozoa capacitated with more potent proteasomal inhibition conditions (100 µM MG132); there were 35.68% of the lactadherin-redistributed spermatozoa in the capacitated-inhibited group compared to 47.65% in the non-inhibited vehicle capacitated group. The WB detection of MFGE8 (Fig. 3C) in 1% TrX-100 extracts from ejaculated and capacitated spermatozoa showed the presence of MFGE8 at 48 kDa in both ejaculated and capacitated sperm, regardless of proteasomal inhibition, while the capacitated sperm extracts showed a unique band at 33 kDa. The 48 kDa isoform of MFGE8 was found to be accumulated (P = 0.009, Fig. S7) in the spermatozoa capacitated with 100 µM MG132 proteasomal inhibition. We also performed WB detection of MFGE8 on sequentially isolated proteins from ejaculated and capacitated spermatozoa, with and without proteasome inhibition, to assess possible redistribution of MFGE8 between soluble and insoluble fractions during sperm IVC (Fig. S8). No such redistribution was observed and the bulk of MFGE8 was extracted up to step 4 by 1% TrX-100.

Another reported proteasome co-purifying protein, the acrosin binding protein (ACRBP, alias sp32) was studied in the same manner as MFGE8. Application of an antibody against the N-terminal part of the ACRBP precursor allowed for the monitoring of the immature, unprocessed form of this protein^31^ and the fate of the N-terminal portion of the protein after its IVC-induced processing. Flow cytometric histogram of immature ACRBP and its N-terminal fragments (Fig. 4A) showed two ACRBP-labeled sperm populations. While the majority of ejaculated spermatozoa were in the population with lower fluorescence intensity, the capacitated spermatozoa were distributed among both populations. From epifluorescence imaging, the N-terminal part of the protein (both the immature protein and presumed degradation products) was localized in the post-acrosomal part of the sperm head and in the sperm tail midpiece (Fig. 4B). In contrast, within the population with higher fluorescence intensity, the N-terminal part of ACRBP accumulated in the midpiece region (Fig. 4B’). With increased proteasomal inhibitor concentration, such accumulation of ACRBP in the midpiece increased as well. After IVC without proteasomal inhibition, 36.55% of spermatozoa were present in the population with ACRBP accumulation in the midpiece region, while 53.48% (P = 0.087 when compared to vehicle control, Fig. S9) of capacitated sperm with mild inhibiting conditions and 58.68% (P = 0.015 compared to vehicle control, Fig. S9) of capacitated sperm with more potent inhibiting were present in the population with midpiece ACRBP. The WB detection of the N-terminal part of ACRBP found the immature forms of ACRBP at approximately 55 kDa and 65 kDa (red arrows in Fig. 4C) and possible degradation products of ACRBP were detected at 42 kDa, 22 kDa, and 19 kDa. From Fig. 4C it is also noticeable that the mass of both immature ACRBP forms is lower under various IVC conditions when compared with ejaculated spermatozoa. While the 55 kDa isoform was still present in capacitated spermatozoa, the 65 kDa form seemed to be processed during IVC. Densitometric analysis of both forms showed that within the limit of detection, neither of these forms were accumulated under proteasomal inhibition (data not shown). All three forms of degradation products were present in both ejaculated and capacitated spermatozoa regardless of proteasomal inhibition. To exclude the possibility that immature ACRBP and its degradation products are redistributed differentially upon IVC, we performed WB detection of ACRBP on sequentially isolated protein fractions from ejaculated and capacitated spermatozoa, with and without proteasome inhibition (Fig. S10). No such redistribution was observed, as no ACRBP signal was detected following 1% TrX-100 extraction.

Another proteasome co-purifying protein, ADAM5 (A Disintegrin And Metalloproteinase 5), was screened in the same manner as previous two proteins (Fig. 5). Flow-cytometric measurements revealed the presence of two sperm populations (Fig. 5A): the population with lower fluorescence intensity, with the majority of non-capacitated and a portion of capacitated spermatozoa, showed labeling in the post-acrosomal region (Fig. 5B). The second peak with higher fluorescence intensity represented the remaining capacitated sperm population, with redistributed ADAM5 in the post-acrosomal region; however, now covering a larger area (Fig. 5B’) than in the population with lower fluorescence intensity. Proteasomal inhibition did not have a significant effect on ADAM5 redistribution when compared to vehicle control (P = 0.180, Figs 5A and S11). WB detection of ADAM5 (Fig. 5C) revealed two distinct bands, a band of 38 kDa present in both ejaculated and capacitated spermatozoa, and a 32 kDa band, present exclusively in capacitated spermatozoa. Other minor bands detected in both the ejaculated and capacitated sperm fractions migrated at 67 and 49 kDa, and minor bands unique to ejaculated spermatozoa were detected at 21, and 13 kDa. During IVC with proteasomal inhibition, protein bands of 38 and 32 kDa were accumulated (P < 0.001 for both bands, Fig. S11) in capacitated spermatozoa. As was the case of MFGE8 and ACRBP, we also monitored the redistribution of ADAM5 between soluble and insoluble compartments during IVC by WB detection in sequentially extracted proteins (Fig. S12). In ejaculated spermatozoa, ADAM5 signal was predominantly detected in the first four fractions. After IVC, the ADAM5 signal shifted to the last two fractions, clearly indicating the change in solubility, likely a result of sperm compartment redistribution.

The last studied proteasome co-purifying protein was acrosin inhibitor (SPINK2). Flow-cytometric measurements detected the presence of two labeling signatures (Fig. 6A): (i) post-acrosomal labeling (Sign 1), and (ii) labeling in the acrosomal region (Sign 2); the specificity of this labeling was confirmed by incubation of spermatozoa with non-immune rabbit serum (Fig. S2). A majority of ejaculated spermatozoa displayed Sign1, while up to 10% of capacitated spermatozoa showed Sign 2. Proteasomal inhibition during IVC did not have any effect on the redistribution between Sign 1 and Sign 2 (P > 0.999, Fig. S13). We also conducted flow cytometric analysis on the acetone fixed/permeabilized spermatozoa and observed acrosomal labeling in spermatozoa with intact acrosomes (Fig. S14A). We observed a significant decrease in mean fluorescence intensity of SPINK2 in all treatments of capacitated spermatozoa after IVC (P < 0.050, Fig. S15) except 100 µM MG132 treatment (P = 0.139, Fig. S15). Proteasomal inhibition had no significant effect on SPINK2 accumulation during IVC. The WB detection of SPINK2 in the total protein extracts revealed the presence of a typical 12 & 14 kDa band doublet^32^ in both ejaculated and capacitated spermatozoa (Fig. 6C). To confirm the presence of SPINK2 in capacitated spermatozoa, blots had to be overexposed, which precluded densitometric analysis. However, even with the strong proteasomal inhibition (100 µM MG132), SPINK2 processing was not substantially inhibited (Fig. 6C), confirming the results obtained by flow cytometry. Uniquely for this proteasome co-purifying protein, SPINK2 was observed to be sequentially released from ejaculated and capacitated spermatozoa (Fig. 6D). In the ejaculated spermatozoa, SPINK2 was continuously released throughout the five steps of sequential treatment, and the final portion of it was released in the fifth step of extraction (2% AcOH). In the control capacitated spermatozoa, SPINK2 was completely released in the third step (OBG treatment); however, SPINK2 was partially retained in the proteasomally-inhibited capacitated spermatozoa, and this portion was released in the fourth extraction step (TrX-100 treatment). Within the limit of detection, there was no SPINK2 present in the subsequent extraction steps.

### Parallel and sequential sperm protein isolation yield differentially accumulated proteins during IVC with vs. without proteasomal modulation

**Figure 7:**
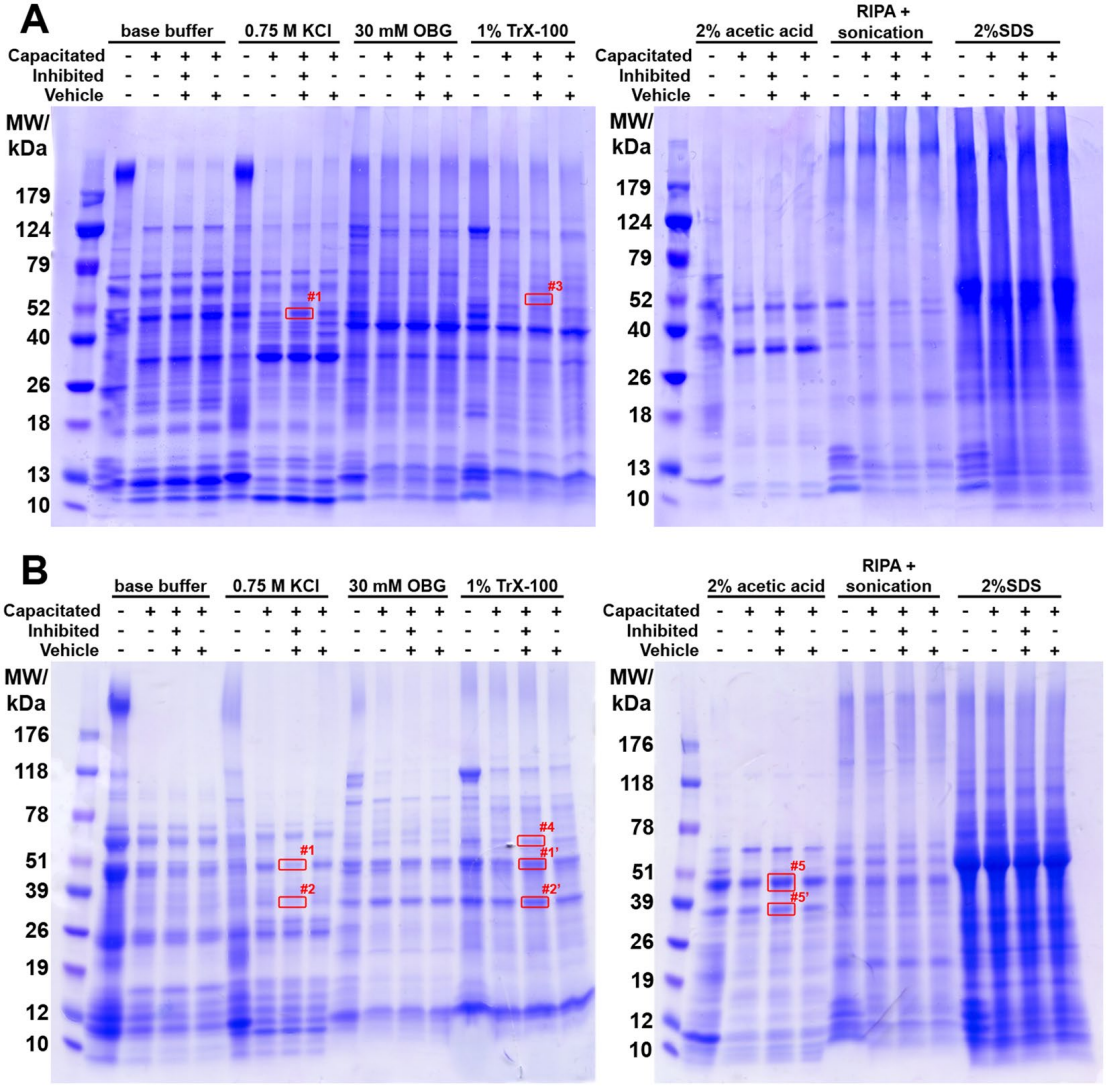
The SDS-PAGE resolved proteins obtained by parallel (**A**) and sequential (**B**) sperm extraction in a compartment-specific manner from ejaculated and *in vitro* capacitated spermatozoa under proteasome permissive/inhibiting conditions (10 μM epoxomicin + 10 μM MG132), including vehicle control (0.2% (v/v) DMSO). Among extraction reagents, n-octyl-β-D-glucopyranoside (OBG), and radioimmunoprecipitation assay (RIPA) buffer were used. Four bands (#1–4), highlighted in red rectangles, were found to be differentially accumulated during capacitation under proteasome inhibiting conditions and were subjected to mass spectrometric (MS) analysis. Bands 5 and 5′ were also found to be accumulated, but not analyzed by MS. Proteins were resolved on a 4–20% gradient gel under reducing conditions, and protein equivalent of 50 million spermatozoa was loaded per single lane. The experiment was done in two replicates; representative electrophoretograms are shown for both parallel and sequential treatments.

In order to identify potential new UPS target proteins during sperm capacitation, we implemented our parallel and sequential isolation protocols, to extract proteins that undergo capacitation-induced changes in solubility and compartmentalization. Proteins extracted from ejaculated spermatozoa and capacitated spermatozoa, under proteasome permissive vs. moderate inhibiting conditions (10 μM epoxomicin + 10 μM MG132), were resolved by SDS-PAGE (Fig. 7), and screened for deferentially accumulated protein bands. In the parallel sperm protein extraction electrophoretogram (Fig. 7A), differentially accumulated protein bands were observed in 0.75 M KCl extract at the molar weight of approximately 49 kDa (band #1) and in the 1% TrX-100 extract at the molar weight approximately 60 kDa (band #3). In the sequential extraction electrophoretogram (Fig. 7B), two differentially accumulated protein bands were observed: The 49 kDa band in the second extraction step (0.75 M KCl) was identical with the 0.75 M KCl extract band in Fig. 7A, and therefore was termed identically (band #1), and the second band at ~35 kDa was designated band #2. These bands were also observed to be differentially accumulated in the fourth treatment step (1% TrX-100), and termed band #1’ and #2’. Bands #1 and #2 were retained in the spermatozoa capacitated under proteasome inhibiting conditions up to fourth extraction step. Another differentially accumulated protein band was observed in the fourth fraction (1% TrX-100) at the molar weight of approximately 58 kDa (band #4). Bands #1-4 were identified by mass spectrometry as summarized in Table 1. Both bands #1 and #2 contained the milk fat globule-EGF factor 8 (MFGE8; aliases p47, lactadherin, SED1), which indeed has two isoforms of corresponding masses^33,34^. Also present in both bands was acrosin (ACR), of which the alpha-heavy chain migrates at 49 kDa and the beta-heavy chain at 34 kDa under reducing conditions^35–38^. Protein present uniquely in band #1 was cathepsin F (CTSF), while the protein in band #2 was zona pellucida-binding protein 2 (ZPBP2). For bands #3 and #4, the commonly identified proteins were: tubulin beta 4B class IVb (TUBB4B), leucyl and cystinyl aminopeptidase/cytosol aminopeptidase (LNPEP); and ATP synthase, H + transporting, mitochondrial F1 complex, alpha subunit 1, cardiac muscle (ATP5A1). Proteins identified uniquely in band #3 were CD46 molecule pseudogene 1/membrane cofactor protein-like (CD46P1) and dihydrolipoamide dehydrogenase/dihydrolipoyldehydrogenase (DLD). Proteins identified uniquely in band #4 were aldehyde dehydrogenase 2 family (ALDH2) and aldehyde dehydrogenase 7 family member A1/alpha aminoadipic semialdehyde dehydrogenase/antiquin (ALDH7A1). Most of the identified proteins in corresponding bands are in agreement with their cDNA predicted molar masses (see Table 1). Differentially accumulated bands were also observed in the fifth step (2% AcOH) at molar weights 49 and 35 kDa, numbered #5 and #5′ respectively. Bands #5 and #5′ were not subjected to mass spectrometric identification, as from the nature of the isolation and apparent molar masses these were presumed to be the heavy chain of α- and β-acrosin, respectively^35^, as confirmed earlier by WB (Fig. 1C).

## Discussion

This study aims to elucidate the role of UPS in boar sperm capacitation. Protein tyrosine phosphorylation and proacrosin/acrosin conversion, as previously reported markers of sperm capacitation^28,29,39^, were utilized to validate our *in-vitro* capacitation (IVC) system, where we observed both an increase in tyrosine phosphorylation (Fig. 1A,B), and proacrosin conversion into acrosin after IVC (Fig. 1C). After assuring robust IVC, we performed proteasome inhibition studies. With the PNA lectin, which specifically binds to saccharide moieties uniquely present on the outer acrosomal membrane (OAM) and is routinely used for monitoring of acrosomal integrity, we observed OAM remodeling during IVC. We identified four different sperm populations based on the PNA-induced fluorescence intensities (Fig. 2). As the majority of ejaculated spermatozoa were present in Pop1, it can be implied that Pop1 represents non-capacitated spermatozoa. As noted previously, spermatozoa that were not used for IVC immediately after semen collection were stored in BTS until the time beginning of IVC experiment. Boar spermatozoa stored in extender media (e.g. BTS, Androhep Plus) undergo capacitation-like changes including modifications in viability, motility, chlortetracycline (CTC) fluorescence patterns, and sperm protein tyrosine phosphorylation^40^. This trend can be observed in Fig. 2 where a higher proportion of spermatozoa from the ejaculated group is present in Pop2 when compared to non-extended ejaculated spermatozoa shown in Fig. S5B,C. It can be therefore assumed that Pop2 represents spermatozoa in the early stages of capacitation. As the sperm capacitation is asynchronous and continuous, it is not surprising that after IVC spermatozoa were distributed among all four populations representing different stages of capacitation. As we already postulated that Pop1 represents non-capacitated spermatozoa and Pop2 most likely represents spermatozoa in early stages of capacitation, we assume in the same manner that Pop3 most likely represents spermatozoa in mid- to late- stages of capacitation. Pop4 could then represent capacitated and/or moribund spermatozoa. Our assumption is supported by an increase in protein tyrosine phosphorylation across these populations (Fig. S5D). This approach allows for the monitoring of the progress and speed of capacitation in individual males or ejaculates. While a majority of spermatozoa from boar shown in Fig. 2 is non-capacitated and early in IVC capacitation, a majority of spermatozoa from another boar shown in Fig. S5B is already capacitating in early- and mid- stages. It is also obvious that spermatozoa used in Fig. S5B capacitated relatively faster than those used in Fig. 2, implying boar-to-boar capacitation rate differences. This becomes even more evident in the third boar (Fig. S5C), indicating capacitation rate differences between individual boars. Regardless of the progress and the speed of capacitation, the progress of OAM remodeling during IVC was hindered by proteasomal inhibitors in all boars (Figs 2 and S5B,C), in agreement with the reported lack of progesterone-induced acrosomal exocytosis in spermatozoa capacitated with proteasomal inhibitors^26^. Interestingly, the ability to achieve Pop4 state was not affected by proteasomal inhibition. We speculate that Pop4 (±10%) after IVC might be the subpopulation of spermatozoa previously identified as Pop2 before IVC. This would imply that the UPS regulates events responsible for OAM remodeling during the early stages of capacitation. It is thought that acrosomal remodeling during capacitation is mainly dependent on protein kinase A (PKA) activity and acrosomal exocytosis is PKC-dependent and that the two events have an intricate cross-talk regulated by PKA-induced activation of phospholipase C (PLC). During sperm capacitation, the activation of PLC by PKA allows for F-actin formation and later activates PKC^41^, a process that could also be regulated by UPS. Beside Tyr phosphorylation, Ser/Thr protein phosphatase inactivation is also important during capacitation^42^. Although we did not see a significant inhibition of Tyr phosphorylation under IVC conditions affecting proteasomal activity, the regulation of Ser/Thr phosphorylation was not assessed and should be examined in further studies. Hindered remodeling of OAM in the proteasomally-inhibited, IVC spermatozoa may prevent close apposition of apical PM and OAM in preparation for multi-point membrane fusion^43^, and desensitizing such spermatozoa to the inducers of acrosomal exocytosis. Further studies are required to prove this hypothesis.

To determine whether and in what manner the proteins previously reported to co-purify with the 26S proteasome were processed during sperm IVC, we implemented IBFC and WB analysis after IVC under the proteasome permissive and inhibiting conditions (Figs 3–6). The redistribution of lactadherin MFGE8 (aliases p47, SED1) from the apical ridge region of the ejaculated spermatozoa to the entire acrosomal region of capacitated spermatozoa was observed in accordance with previously reported results as well as the presence of two different forms of lactadherin in spermatozoa^34,44–47^. Furthermore, MFGE8 can be isolated from OAM-matrix complex of bovine spermatozoa after IVC^45^. We found that the proteasome participates in events linked to the redistribution and degradation of MFGE8, which was hindered by proteasomal inhibitors causing the accumulation of the 48 kDa isoform (Fig. 3). Authors in^45^ propose that the translocation of bovine MFGE8 in the detergent-insoluble acrosomal matrix fraction may trigger a cascade of events priming the spermatozoa for capacitation and/or acrosomal exocytosis. We suspect that the redistribution of MFGE8 in porcine spermatozoa follows the same pattern; however, our sequential isolation technique (Fig. S8) was not selective enough to prove this hypothesis. Collectively, the OAM remodeling and MFGE8 redistribution during capacitation may be another reason why proteasomally inhibited spermatozoa are unable to undergo acrosomal exocytosis, as reported by Sanchez *et al*. and Chakravarty *et al*.^26,48^. MFGE8 was recently proposed to be a candidate oviductal cell receptor for Lewis X trisaccharide on porcine spermatozoa^34^. Therefore, it seems reasonable that MFGE8 redistribution during capacitation conveys the ability of capacitated spermatozoa to detach from the oviductal sperm reservoir-epithelium. Consistent with our findings, the proposed role of MFGE8 in relation to UPS is as an anchor, substrate or interactor for the sperm 26 S proteasome^25^.

The acrosin binding protein ACRBP (syn. sp32) is synthesized as a 61 kDa precursor protein^31^, that undergoes post-translational processing through the removal of the N-terminal half; the carboxyl-terminal sequence corresponds to mature 32 kDa form^31,49^. Antibodies specific to the N-terminal sequence of ACRBP precursor allowed us to monitor the fate of the immature, intact and partially degraded ACRBP species, reported earlier as proteasome co-purifying proteins^25^. To our knowledge, there has been no research done on immature ACRBP and its degradation products in boar spermatozoa. We confirmed the presence of ACRBP precursor in ejaculated spermatozoa by detecting the signature 65/54 kDa doublet^31,50^. The long, N-terminal part containing ACRBP precursor is localized in the post-acrosomal region and the midpiece of both ejaculated and capacitated spermatozoa. With no previous studies addressing immature ACRBP to compare our results with, the function and involvement of the long, N-terminal part containing ACRBP precursor in the sperm capacitation and fertilization, in general, remains elusive. Nevertheless, we confirmed that the proteasome is involved in the processing of ACRBP N-terminus, at least in the midpiece region (Fig. 4). To our knowledge, this is the first report on the fate of ACRBP precursor after capacitation. While the carboxy-terminal domain of mature sp32 is responsible for acrosin binding and activation during capacitation^31^, the flagellar N-terminus containing species is most likely regulating sperm motility and hyperactivation. It will be further investigated whether the N-terminus containing ACRBP precursor might have a unique function in spermatozoa.

A disintegrin and metalloprotease domain-containing protein 5 (ADAM5) is a membrane-anchored surface protein, synthesized as large precursors (98 kDa) and processed to 44- to 49-kDa products (49 kDa for ADAM5) during epididymal passage^51^. We detected ADAM5 in Triton X-100 extracts of ejaculated boar spermatozoa predominantly at the molar mass of 38 kDa and to a lesser extent at 49 kDa (Fig. 5C), which is in accordance with Silva *et al*.^34^, suggesting further processing or degradation of ADAM5 upon contact with the seminal plasma. In addition, ADAM5 seems to be further processed during the course of IVC, where we detected two distinct forms of 38 kDa and 32 kDa. Such processing varies between individual boars and is accompanied by ADAM5 spreading across the post-acrosomal region (Fig. 5B’). Proteins localized to the post-acrosomal region have generally low solubility, as is the case of ADAM5, becoming even less soluble after IVC (Fig. S12). While this ADAM5 expansion does not seem to be hindered when proteasomal inhibitors are introduced into IVC medium, ADAM5 fluorescence is higher in spermatozoa capacitated with proteasomal inhibition when compared to vehicle control, suggesting the contribution of the proteasome to the capacitation-induced processing of ADAM5. Furthermore, a decrease in ADAM5 solubility was hindered in spermatozoa capacitated under proteasomal inhibition, suggesting indirect UPS participation in the protein redistribution between the soluble and extraction resistant sperm compartments, further supported by ADAM5 accumulation under proteasomal inhibition (Fig. 5C). As in the case of MFGE8, ADAM5 is a candidate sperm adhesion molecule to the oviductal epithelium^34^, implying participation in the formation of the oviductal sperm reservoir and UPS-modulated sperm detachment from it. We also demonstrate that ADAM5 is redistributed during sperm IVC and proteasome participates in this event. The nature and the purpose of this redistribution, as well as ADAM5 processing during IVC, will be the subject of further studies. We can speculate that ADAM5 redistribution, accompanied by the decrease in ADAM5 solubility, may be a preparative step for sperm-oolemma adhesion and fusion. ADAM5 may then participate in sperm-oolemma adhesion as an individual molecule or in a previously described trimer with ADAM2 and ADAM3^51^.

Kazal type 2/acrosin inhibitor (SPINK2), a serine peptidase inhibitor, was extensively studied and characterized in previous studies^52–58^. Two forms of SPINK2 were reported; seminal plasma SPINK2 (SPAI) and sperm associated SPINK2 (SAAI), slightly differing in amino acid sequence and glycosylation. We previously demonstrated that the subunit PSMD4 of the 19S proteasomal regulatory complex of the 26S proteasome co-immunoprecipitates with SAAI/SPINK2, as the first line of evidence that SAAI activity is controlled by the UPS^32^. In the present study, we used antibodies raised against the sperm-associated inhibitor/SAAI isoform of SPINK2. In ejaculated spermatozoa, we detected SPINK2 at 12 kDa, as anticipated based on amino acid sequence and glycosylation (Fig. 6C), which is in agreement with previous results^52,53^. We could confirm that the majority of SPINK2 was released during IVC, as this removal is essential for fertilization^59^. No significant retention of SPINK2 was observed in capacitated spermatozoa with strong proteasomal inhibition (Fig. 6C). However, a portion of SPINK2 was retained in proteasomally-inhibited, IVC spermatozoa during sequential extraction up to the fourth step, rather than in the third step as was the case in the absence of proteasomal inhibition (Fig. 6D). In IBFC, the labeling of the acrosomal area, as reported in previous studies^32,56^, was only present in ~10% of formaldehyde-fixed, capacitated spermatozoa, and the rest of capacitated spermatozoa, as well as all ejaculated spermatozoa, were labeled in the post-acrosomal region (Fig. 6B). This staining was consistent with both anti-seminal plasma and anti-sperm SPINK2 antibodies (data not shown); non-immune serum control ruled out non-specific labeling. With this in mind, we designated two differently labeled populations as signature 1 and 2. However, as in the case of Western blot detection, we were unable to find a significant difference between proteasomally-inhibited and non-inhibited IVC spermatozoa. The last-ditch effort to implicate UPS in SPINK2 degradation by flow cytometric detection of SPINK2 in the acetone fixed/permeabilized spermatozoa (Fig. S14) yielded labeling of the acrosomal area in spermatozoa with intact acrosomes, which decreased significantly in all IVC spermatozoa, except those capacitated with 100 µM MG132 (Fig. S15). Similarly, we did not observe a significant accumulation of SPINK2 during IVC in the proteasomally-inhibited, IVC spermatozoa. Taken together, these data imply only marginal participation of proteasome in SPINK2 processing during capacitation and adds to our previously reported finding that E1/UBE1 is involved the UPS dependent release/degradation of SPINK2 during boar sperm capacitation^24^.

To identify additional proteins processed or regulated by UPS during sperm capacitation, we applied differential sperm compartment fractionation and protein isolation from ejaculated spermatozoa, and spermatozoa capacitated under proteasomal activity-permissive or inhibiting conditions. We identified several differentially accumulated protein bands that were submitted for the mass spectrometric identification. The most abundant proteins are presented in Table 1, with more details in Supplementary Data. We are fully aware of the limitations of 1DE approach which leads to identifying multiple proteins with the approximately same molar mass within one band, though the robustness of this approach is improved by our differential extraction procedures, as well as sequential protein isolation protocol to isolate proteins according to their solubility and compartmentalization. Under mild extraction conditions, the increase in protein amount could be as well due to a change in solubility in the respective buffer as a result of its translocation between sperm compartments or degradation, rather than being representative of a change in the amount of the said protein. Considering these drawbacks, the identified proteasome targeted proteins need to be further studied to confirm UPS participation in their processing during sperm capacitation. Some of the identified proteins (MFGE8 and acrosin) were already studied in the present study.

**Table 1:** Summary of MS/MS protein identification analysis of four bands (#1–4) from Fig. 7, found to be differentially accumulated during capacitation under proteasome inhibiting conditions.

Protein annotation	NCBI gi #	HUGO name (Symbol)	Aliases	Ion Score	% Coverage	Nominal MW (kDa)	Protein score
**#1, 49 kDa protein band**							
Lactadherin precursor	172072653	milk fat globule-EGF factor 8 protein (MFGE8)	BA46, EDIL1, hP47, HsT19888, “lactadherin”, MFG-E8, OAcGD3S, SED1, “sperm surface protein hP47”	738	36	49.0	1159
Acrosin precursor (EC 3.4.21.10)	97535584	acrosin (ACR)	“acrosin light and heavy chain prepropeptide”, “preproacrosin”	346	24	46.0	642
Predicted: Cathepsin F isoform X1 (EC 3.4.22.41)	335281454	cathepsin F (CTSF)	CATSF, CLN13	310	16	54.7	295
**#2, 35 kDa protein band**							
Lactadherin precursor	172072653	milk fat globule-EGF factor 8 protein (MFGE8)	BA46, EDIL1, hP47, HsT19888, “lactadherin”, MFG-E8, OAcGD3S, SED1, “sperm surface protein hP47”	324	22	49.0	207
Malate dehydrogenase precursor (EC 1.1.1.37)	164541	malate dehydrogenase 1 (MDH1)	“malate dehydrogenase 1, NAD (soluble)”	223	23	32.2	187
Beta-Acrosin, chain A (EC 3.4.21.10)	11513874	acrosin (ACR)	“acrosin light and heavy chain prepropeptide”, “preproacrosin”	210	18	29.7	287
Predicted: zona pellucida-binding protein 2 isoform X1	545858201	zona pellucida binding protein 2 (ZPBP2)	MGC41930, ZPBPL	195	19	38.0	81
**#3, 60 kDa protein band**							
Predicted: Cytosol aminopeptidase (EC 3.4.11.1)	927165791	leucyl and cystinyl aminopeptidase (LNPEP)	Leucyl aminopeptidase, Cytosol aminopeptidase, CAP, “cystinyl aminopeptidase”, IRAP, P-LAP, “placental leucine aminopeptidase”, PLAP	508	34	56.4	452
Tubulin beta-4b chain	335281298	tubulin beta 4B class IVb (TUBB4B)	Beta2, “class IVb beta-tubulin”	285	17	50.3	331
Predicted: membrane cofactor protein-like	350588758	CD46 molecule pseudogene 1 (CD46P1)	“CD46 molecule, complement regulatory protein pseudogene”, “CD46 molecule, complement regulatory protein pseudogene 1”, CD46P, MCPL, “membrane cofactor protein-like (CD46-like, trophoblast-lymphocyte cross-reactive antigen-like)”	271	19	49.6	421
Dihydrolipoyl dehydrogenase, mitochondrial precursor (EC 1.8.1.4)	47522940	dihydrolipoamide dehydrogenase (DLD)	“dihydrolipoamide dehydrogenase (E3 component of pyruvate dehydrogenase complex, 2-oxo-glutarate complex, branched chain keto acid dehydrogenase complex)”, Glycine cleavage system L protein, GCSL, LAD, dihydrolipoyl dehydrogenase, LDP-Glc, LDP-Val, dehydrolipoate dehydrogenase, diaphorase, dihydrolipoamide:NAD + oxidoreductase, dihydrolipoic dehydrogenase, dihydrothioctic dehydrogenase, lipoamide dehydrogenase (NADH), lipoamide oxidoreductase (NADH), lipoamide reductase, lipoamide reductase (NADH), lipoate dehydrogenase, lipoic acid dehydrogenase, lipoyl dehydrogenase, protein-6-N-(dihydrolipoyl)lysine:NAD + oxidoreductase	238	14	54.7	217
ATP synthase subunit alpha, mitochondrial	297591975	ATP synthase, H + transporting, mitochondrial F1 complex, alpha subunit 1, cardiac muscle (ATP5A1)	ATP5A, ATP5AL2, ATPM, hATP1, OMR, ORM	284	14	59.8	188
**#4, 58 kDa protein band**							
ATP synthase subunit alpha, mitochondrial	297591975	ATP synthase, H + transporting, mitochondrial F1 complex, alpha subunit 1, cardiac muscle (ATP5A1)	ATP5A, ATP5AL2, ATPM, hATP1, OMR, ORM	575	22	59.8	570
Mitochondrial aldehyde dehydrogenase 2 (EC:1.2.1.3)	187370719	aldehyde dehydrogenase 2 family (mitochondrial) (ALDH2)	ALDH class 2, ALDH-E2, ALDHI	394	17	57.3	247
Predicted: cytosol aminopepdidase (EC 3.4.11.1)	927165791	leucyl and cystinyl aminopeptidase (LNPEP)	Leucyl aminopeptidase, Cytosol aminopeptidase, CAP, “cystinyl aminopeptidase”, IRAP, P-LAP, “placental leucine aminopeptidase”, PLAP	284	17	56.4	254
Predicted: alpha aminoadipic semialdehyde dehydrogenase (EC:1.2.1.3)	927105880	aldehyde dehydrogenase 7 family member A1 (ALDH7A1)	“26 g turgor protein homolog”, “alpha-AASA dehydrogenase”, “alpha-aminoadipic semialdehyde dehydrogenase”, “antiquitin 1”, Betaine aldehyde dehydrogenase (EC:1.2.1.8), “delta1-piperideine-6-carboxylate dehydrogenease”, EPD, “P6c dehydrogenase”, PDE	272	10	59.0	309
Tubulin beta-4b chain	335281298	tubulin beta 4B class IVb (TUBB4B)	Beta2, “class IVb beta-tubulin”	252	17	50.3	252

For the search against NCBInr Mammalian protein database, an individual MS/MS Ion Score > 44 indicated identity or extensive homology, P < 0.05. The total ion score for a protein assignment is the sum of all the highest statistically significant ion scores for matched peptide MS/MS. Only the top three to five matches with the highest statistical credibility per individual band are shown. Relative protein abundances, within each band, are represented as protein score.

In conclusion, the above studies document the participation of UPS in the process of sperm capacitation. Proteasomal activity during the course of capacitation thus appears necessary for processing of several previously reported 26S proteasome co-purifying proteins. Multiple new proteins have been identified which are likely regulated by UPS during sperm capacitation either directly or through indirect mechanisms. Further studies will determine how they are processed and how these changes contribute to the progression of sperm capacitation.

## Materials and Methods

### Statement of Scientific Rigor

All experiments in this article have been replicated with appropriate controls and consistent results between replicates. Readers are strongly encouraged to peruse Supplemental Data File and contact corresponding authors should concern arise about the repeatability.

### Antibodies and reagents

Proteasomal inhibitors epoxomicin (cat # BML-PI127) and MG132 (cat # BML-PI102) were purchased from ENZO Life Sciences, Farmingdale, NY, USA. Beltsville thawing solution (BTS) boar semen extender supplied with gentamicin was purchased from IMV Technologies, Maple Grove, MN, USA. Halt^TM^ Protease and Phosphatase inhibitor (cat # 78443) was purchased from ThermoFisher Scientific, Rockford, IL, USA. PAGEr^TM^ Gold gels (cat # 59545) and Prosieve protein colored markers were purchased from Lonza Rockland Inc., Rockland, ME, USA. Bradford protein assay dye (cat # 5000006) was purchased from Bio-Rad, Hercules, CA, USA. PVDF Immobilon Transfer Membrane and Luminata Crescendo Western HRP Substrate were bought from Millipore Sigma, Burlington, MA, USA. Sequencing grade modified trypsin (cat # V5111a) was purchased from Promega Corporation, Madison, WI, USA. All other chemicals used in this study were purchased from Sigma-Aldrich, St. Louis, MO, USA.

Mouse monoclonal anti-phosphotyrosine antibody, clone 4G10 (cat #05-321) was bought from Millipore Sigma, Burlington, MA, USA. Mouse monoclonal anti-acrosin antibody, Acr-2 was a kind gift from Dr. Jana Pěknicová, Institute of Biotechnology of the Czech Academy of Sciences^30,60^ (also commercially available, cat # MA1-19180, ThermoFisher Scientific, Rockford, IL, USA). Rabbit polyclonal anti-acrosin binding protein (ACRBP) antibody (cat # PA5-38735) was bought from Invitrogen, Carlsbad, CA, USA. Rabbit polyclonal anti-ADAM5 antibody (cat # ab101143) was bought from Abcam, Cambridge, United Kingdom. Rabbit polyclonal anti-sperm associated, acrosin inhibitor (SPINK2) antibody^53,61^ and anti-P47 lactadherin antibody, clone 1H9^47^ were raised in-house. Goat anti-mouse IgG (cat # 31430) and goat anti-rabbit IgG (cat # 31460), HRP secondary antibodies were purchased from ThermoFisher Scientific, Rockford, IL, USA. Goat anti-mouse IgG, TRITC (cat # A16071) and goat anti-rabbit IgG, Cy5 (cat # A10523) secondary antibodies were purchased from Invitrogen, Carlsbad, CA, USA. PNA lectin, AF488, and DAPI were purchased from Molecular Probes, Eugene, OR. USA.

### Semen processing and *in vitro* sperm capacitation (IVC) with proteasomal modulation

All studies involving vertebrate animals were completed under the strict guidance of an Animal Care and Use protocol approved by the Animal Care and Use Committee (ACUC) of the University of Missouri. Fresh boar spermatozoa were collected on a weekly basis from three healthy, non-transgenic fertile boars used for routine *in vitro* fertilization trials with high blastocyst yield, and the sperm-rich fraction was used for the study purposes. Concentration and motility of ejaculates were evaluated by conventional spermatological methods under a light microscope. Sperm concentration was measured by hemocytometer (ThermoFisher Scientific) and ranged from 250 to 350 million/mL; only ejaculates with >80% motile spermatozoa and <20% morphological abnormalities were used for the study. Ejaculates were free of contaminants other than the expected minimal content of cytoplasmic droplets, thus not necessitating gradient purification. Spermatozoa were separated from seminal plasma (2,000 RPM ~ 400 *g*, 10 min; ThermoFisher Scientific). Spermatozoa were either diluted tenfold with pre-warmed (within 2 °C of semen temperature at collection) Beltsville thawing solution (BTS) boar semen extender supplied with gentamicin (IMV Technologies) and allowed to cool down to room temperature for further use, or used fresh directly for capacitation studies.

Fresh, non-extended spermatozoa, separated from seminal plasma, were capacitated as described previously^22^. Spermatozoa were treated during IVC: i) with no proteasomal inhibition in order to monitor unaltered (by proteasomal inhibitors) sperm IVC; ii) with proteasomal inhibition to monitor sperm IVC under proteasome inhibiting conditions, either supplemented with 10 μM MG132 and 10 μM Epoxomicin (both ENZO Life Sciences, mild inhibiting conditions) in Dimethyl Sulfoxide (DMSO) or 100 μM MG132 in Ethanol (EtOH, strong inhibiting conditions); and iii) vehicle control in order to account for potential vehicle interference with sperm IVC, which was either 0.2% (v/v) DMSO for MG132 and Epoxomicin, or 0.2% (v/v) EtOH for MG132 alone, the inhibitors used here were previously characterized^62^. Uniquely for outer acrosomal membrane remodeling study, 0.2% (v/v) DMSO was used as the vehicle for both 10 μM MG132 and 10 μM Epoxomicin and 100 µM MG132 proteasomal inhibition. Sperm samples after IVC were washed from BSA and used for protein extraction and flow cytometric quantification.

### Image-based flow cytometry (IBFC)

Ejaculated spermatozoa, stored in BTS medium at room temperature; and fresh, non-extended capacitated spermatozoa with or without proteasomal inhibitors or vehicle solutions were processed as previously described^22^. Briefly, spermatozoa were washed three times with warm phosphate-buffered saline (PBS; 137 mM NaCl, 2.7 mM KCl, 10 mM Na_2_HPO_4_, 1.8 mM KH_2_HPO_4_, pH = 7.4), fixed in 3.7% formaldehyde and permeabilized in 0.1% TrX-100 in PBS (PBST). Apart from formaldehyde fixation, fresh, washed spermatozoa were permeabilized with ice-cold acetone for 15 min at −25 °C for acrosin inhibitor (SPINK2) labeling and washed three times with PBST. Approximately 20 million spermatozoa were blocked with PBST supplemented with 5% normal goat serum (NGS). All the antibodies used for flow cytometric studies were previously characterized. Primary antibodies used were as follows: anti-acrosin binding protein (ACRBP, 1:100 dilution; PA5-38735, Invitrogen), anti ADAM5 (1:100 dilution; ab101143, Abcam), anti-sperm associated, acrosin inhibitor (SPINK2; 1:200 dilution; [27]), anti-P47 lactadherin, clone 1H9 (1:200 dilution)^47^, and anti-phosphotyrosine, clone 4G10 (1:100 dilution; 05-321, Millipore Sigma); all diluted in PBST supplemented with 1% NGS. Primary antibodies were added to sperm sample tubes and incubated overnight at 4 °C. For negative controls, non-immune mouse and rabbit sera of comparable globulin concentrations were used instead of primary antibodies and processed in the same fashion. The following morning, spermatozoa were washed twice with PBST with 1% NGS, and appropriate species-specific secondary antibodies such as goat anti-mouse conjugated to tetramethylrhodamine isothiocyanate (GAR-TRITC; Invitrogen) and goat anti-rabbit conjugated to Cyanine5 (GAR-Cy5; Invitrogen) were diluted 1:150 in PBST with 1% NGS, and allowed to incubate for 40 min at room temperature. For acrosome integrity assessment, peanut agglutinin lectin conjugated to Alexa Fluor 488 (PNA-AF488, 1:2,500 dilution; Molecular Probes) was used, and 4’,6-Diamidino-2-Phenylindole Dilactate (DAPI), a DNA stain (1:1500 dilution; Molecular Probes) was used as a reference and nuclear contrast stain. Both PNA-AF488 and DAPI were mixed and coincubated with secondary antibodies. After incubation with secondary antibodies, spermatozoa were washed twice with PBST with 1% NGS. Prior to performing IBFC, the sample aliquots were checked for fluorescence labeling under a Nikon Eclipse 800 epifluorescence microscope (Nikon Instruments, Melville, NY, USA). The final concentration was adjusted to 20 mil spermatozoa per 100 μl.

For outer acrosomal remodeling studies, the washed, non-fixed spermatozoa were used immediately after IVC, permeabilized with PBST for 30 minutes at room temperature, incubated with PNA-AF488 and DAPI for 40 minutes at room temperature, washed twice with PBST and flow cytometry acquisitions were performed. The study was repeated with formaldehyde-fixed spermatozoa.

The fluorescently labeled samples were measured with an Amnis FlowSight Imaging Flow Cytometer (EMD Millipore Corp., Seattle, WA, USA) fitted with a 20X microscope objective as described previously^12,22^. The instrument parameters and detailed settings are presented in Supplementary Material. Samples were analyzed using five lasers concomitantly: a 405-nm line, 488-nm line, 561-nm line, 642-nm line a 785-nm line (side scatter). A total of 10,000 events were collected per sample, and data were analyzed using IDEAS® software (Version 6.2.64.0; Amnis-Millipore). Single-cell population gate was used for histogram display of mean pixel intensities by frequency for collected channels. Intensity histograms of individual channels were then used for drawing regions of subpopulations with varying intensity levels and visual confirmation. The intensity of DAPI was used for histogram normalization among different treatment groups. Fluorescently positive debris was excluded by applying masks. The Feature Finder tool was utilized to identify the most relevant optical/morphometric feature of sub-populations difference, where mean pixel intensities were not sufficiently distinctive. Negative controls of normal mouse and normal rabbit sera are included in Suppl. Figs 1 and 2, respectively.

### Parallel and sequential isolation of sperm proteins

Ejaculated spermatozoa stored in BTS extender and all three sperm treatment groups after IVC (proteasomally-inhibited, non-inhibited and vehicle control) were washed three times with Tris-buffered saline (TBS; 50 mM TRIS∙HCl, pH = 7.4, 137 mM NaCl) and used for protein extractions.

The following treatments were applied to accomplish extractions in a solubility and compartmentalization-specific manner: TBS as a base buffer/wash for all the following reagents); 0.75 M KCl, 30 mM n-octyl-β-D-glucopyranoside (OBG), 1% (v/v) Triton X-100 (TrX-100), 2% (v/v) acetic acid (2% AcOH), sonication in modified RIPA buffer (1% (v/v) TrX-100, 1% (w/v) sodium deoxycholate, 0.1% (w/v) SDS, 1 mM DTT, 2 mM EDTA), and SDS-PAGE loading buffer^63^. Approximately 200 million spermatozoa were used per extraction, which was conducted by adding 100 μl of the extraction reagent and protease and phosphatase inhibitors (ThermoFisher Scientific). For the first five treatments, sperm pellets were resuspended and incubated on ice for 30 min with periodic agitation. For the sonication extraction, sperm pellets were sonicated on ice for 1 min with 30% amplitude (Branson Ultrasonics, Danbury, CT, USA). For SDS extraction, sperm pellets were boiled for 5 min. Spermatozoa were spun off (500 *g*, 4 °C, 10 min), and extracts were stored in −25 °C for further analysis.

For sequential (stepwise) isolation of proteins, spermatozoa were treated with selected isolation reagents in the order of ascending strength as follows: TBS was added to each pellet in the first step, allowed to incubate on ice for 30 min, spun off and the extracts were saved. In the second step, pellets were treated with 0.75 M KCl, allowed to incubate on ice for 30 min, spun, and separated into extract and pellet, washed once with cold TBS, and reused in the third step for extraction with 30 mM OBG (30 min incubation on ice). The fourth step was treatment with 1% TrX-100, and the fifth step with 2% AcOH (both 30 min incubation on ice). After the fifth step, modified RIPA buffer was added and spermatozoa were sonicated on ice (60 sec, 30% amplitude; Branson Ultrasonics). Lastly, sperm pellets were boiled in SDS-PAGE loading buffer (seventh step). For parallel (individual) extractions, all seven treatments were done at the same time for all four sperm populations (ejaculated, capacitated, proteasomally-inhibited, vehicle control).

### SDS-PAGE and Western blotting (WB)

Sperm protein extracts, other than those obtained with the SDS sample buffer, were mixed with 4 x concentrated SDS-PAGE loading buffer in the ratio of 3:1, boiled for five minutes and briefly spun at 5,000 *g*. Total protein equivalent of 50 million spermatozoa was loaded per single lane. SDS-PAGE was carried out on 4–20% gradient gels (Lonza) as previously described^25^. The molecular masses of separated proteins were estimated using prestained Prosieve protein colored markers (Lonza) run in parallel. After SDS-PAGE, gels were stained with Coomassie brilliant blue (CBB) R-250 for protein visualization and the electrophoretogram was analyzed for deferentially accumulated protein bands using Image Lab^TM^ Software (ver. 5.2.1, Bio-Rad, Hercules, CA, USA).

For WB detection, 20 μg of protein (concentration determined by Bradford assay^64^) was used per single lane. After SDS-PAGE, proteins were electrotransferred onto a PVDF Immobilon Transfer Membrane (Millipore Sigma) using an Owl wet transfer system (ThermoFisher Scientific) at a constant 50 V for 4 h for immunodetection^5,25^, according to the method described by Towbin *et al*.^65^. Residual gels after electro-transfer were stained with CBB R-250 for protein load control. The PVDF membranes (Millipore Sigma) with the transferred proteins were blocked with 10% (w/v) non-fat milk in TBS with 0.1% (v/v) Tween 20 (TBST) and incubated with the following primary antibodies: anti-acrosin binding protein (ACRBP, 1:2,000 dilution; PA5-38735, Invitrogen), anti-ADAM5 (1:8,000 dilution; ab101143, Abcam), anti-sperm associated, acrosin inhibitor (1:8,000 dilution; SPINK2^53,61^), anti-P47 lactadherin (1:500 dilution; clone 1H9^47^), anti-phosphotyrosine, clone 4G10 (1:2000 dilution; 05-321, Millipore Sigma), and anti-acrosin Acr-2 (1:250 dilution)^60^, overnight. The membranes were then incubated with an appropriate species-specific secondary antibody, such as the HRP-conjugated goat anti-rabbit (GAR-IgG-HRP) or anti-mouse (GAM-IgG-HRP) antibodies (1:10,000 dilution; Invitrogen). The membranes were reacted with chemiluminescent substrate (Luminata Crescendo Western HRP Substrate; Millipore Sigma) and blots were screened with ChemiDoc Touch Imaging System (Bio-Rad, Hercules, CA, USA), to record the protein bands, and analyzed by Image Lab^TM^ Software (ver. 5.2.1, Bio-Rad, Hercules, CA, USA). Membranes were stained either with Ponceau S following electrotransfer or with CBB R-250 after chemiluminescence detection for protein load control.

### Statistical analysis

In order to optimize experimental protocol, three pilot trials (for flow cytometry) and two pilot trials (for Western blotting) were done to establish optimal reagent conditions. Four independent replicates with optimized conditions and consistent results were conducted afterward and used for statistical analysis unless stated otherwise. Each data point is presented as the mean ± SD. Datasets were tested for normal distribution by using Shapiro-Wilk normality test and processed using one-way analysis of variance (ANOVA) using the GraphPad Prism 7.03 (GraphPad Software, Inc., La Jolla, CA, USA) in a completely randomized design. Sidak’s multiple comparison test was used to compare mean values of individual treatments with a 95% confidence interval. A value of P < 0.05 was considered statistically significant.

### Proteomics

Parallel and sequential boar sperm extracts were resolved on 4–20% gradient gels (Lonza) and stained with CBB R-250. The bands of interest were excised carefully from the CBB-stained gel, destained, reduced with DTT, alkylated with iodoacetamide, and trypsinized with TPCK-treated porcine trypsin (V5111a, Promega) overnight at 4 °C. The digested solutions were recovered from the gel cutouts and transferred to Axygen MAXYMum Recovery microtubes (Corning Inc., Corning, NY, USA). The gel cutouts were extracted twice, pooled, and lyophilized dry. The dried digests were reconstituted in 30/70/1 (v/v/v) acetonitrile/water/99% formic acid, and analyzed by Nano LC-Nanospray quadrupole time-of-flight MS plus MS/MS on an Agilent 6520 A QTOF mass spectrometer (Agilent Technologies, Inc., Santa Clara, CA, USA). The “MS plus MS/MS” data were analyzed with the “Find Compounds by Auto MS/MS” program in the Agilent Mass Hunter software (version B.04.00) suite. Nanospray-QTOF MS spectra peak lists were obtained for the spectra after internal recalibration using trypsin autolysis fragment masses, computer baseline correction, noise removal, and peak deisotoping. The threshold for generating peak lists was set to 1% of the maximum observed peak area. Data were exported in Mascot Generic Format (.mgf) for submission to an in-house copy of Matrix Science’s Mascot program (www.matrixscience.com, version 2.4). Database searches were performed against the NCBInr Mammalian Protein Databases (last updated June 30, 2016; 3,639,811 sequences) and adjusted for trypsin digestion with initial 2 missed cleavage sites, fixed modification by carbamidomethylation, and variable modification by methionine oxidation and pyroglutamate formation at peptide N-terminal glutamic acid. The precursor ion mass error tolerance was ±10 ppm and the MS/MS fragment ion mass error tolerance was ±0.1 Da. Total ion and emPAI scores^66^ were used to identify highly probable matches with known amino acid sequences.

### Summary sentence

The activity of sperm ubiquitin-proteasome system modulates the events of sperm capacitation, including the remodeling of the sperm acrosome and shedding of the sperm surface glycoproteins.

## Supplementary information

Supplementary Information

## Data Availability

All data generated or analysed during this study are included in this published article (and its Supplementary Information Files).
