# Supplementary material for: Compartmentalization of the proteasome-interacting proteins during sperm capacitation
Source: Sci Rep. 2019 Aug 29;9:12583. doi: 10.1038/s41598-019-49024-0 (PMC6715765; doi:10.1038/s41598-019-49024-0)
Supplement: Supplementary file 1 — Supplementary Information [file 41598_2019_49024_MOESM1_ESM.pdf]

Compartmentalization of the proteasome-interacting proteins during sperm capacitation

Michal Zigo[a,b,*], Pavla Manaskova-Postlerova[b,c], Vera Jonakova[b], Karl Kerns[a], Peter Sutovsky[a,d]

[a]Division of Animal Sciences, University of Missouri, Columbia, MO 65211;
[b]Laboratory of Reproductive Biology, Institute of Biotechnology of the Czech Academy of Sciences, Vestec, Czech Republic 25250;
[c]Department of Veterinary Sciences, Faculty of Agrobiology, Food and Natural Resources, Czech University of Life Sciences, Prague, Czech Republic;
[d]Department of Obstetrics, Gynecology & Women's Health, University of Missouri, Columbia, MO 65211

Supplementary material

Materials and Methods

Image Based Flow Cytometry (IBFC) parameters and settings

The fluorescently labeled samples were measured with an Amnis FlowSight Imaging Flow Cytometer (EMD Millipore Corp., Seattle, WA, USA) fitted with a 20X microscope objective (numerical aperture of 0.5) with an imaging rate up to 2,000 events/sec. The sheath fluid was PBS, free of Ca^{2+} or Mg^{2+}. The flow-core size and speed was 10 µm diameter and 66 mm/sec, respectively. Raw images were acquired using INSPIRE® software (Amnis-Millipore). The camera was set to 1.0 µm per pixel of the charged-coupled device. The image display dimension for the field of view was 60 µm and 8 µm depth of the field. Samples were analyzed using five lasers concomitantly: a 405-nm line with intensity set to 50mW; 488-nm line with intensity set to 50mW; 561-nm line with intensity set to 50mW; 642-nm line with intensity ranging from 5 to 20mW and a 785-nm line (side scatter) with intensity set to 5mW. A total of 10,000 events were collected per sample, and the electronic images were compensated for channel crossover by using single-color controls (i.e., DAPI-only; AF488-only; TRITC-only; and Cy5-only labeling of spermatozoa) that were merged to generate a multi-color matrix. The compensation matrix file was then applied to an experimental raw-image file, yielding a color-compensated image file. Data analysis of the raw images was accomplished using IDEAS® software (Version 6.2.64.0; Amnis-Millipore). Displaying spermatozoa using Gradient RMS for the bright field channel allowed the gating of focused spermatozoa. A combined Area × Aspect Ratio scatter plot display was used to gate single-cell events. Single-cell population gate was used for histogram display of mean pixel intensities by frequency for following channels: AF488 (channel 2), TRITC (channel 3), DAPI (channel 7), and Cy5 (channel 11). Intensity histograms of individual channels were then used for drawing regions of subpopulations with varying intensity levels and visual confirmation. The intensity of DAPI (channel 7) was used for histogram normalization among different treatment groups. Appropriate masks were applied to all relevant channels to exclude fluorescently positive debris from features' calculation. The Feature Finder tool was utilized to

[*] To whom correspondence should be addressed: Michal Zigo, Division of Animal Sciences, University of Missouri, Columbia, MO 65211-5300, USA; Tel.: +1 (573) 884-1549; Fax: +1 (573) 882-6827; E-mail address: zigom@missouri.edu, alt. michal_zigo_2000@yahoo.com

identify the most relevant optical/morphometric feature of sub-populations difference, where mean pixel intensities were not sufficiently distinctive.

Supplementary figures

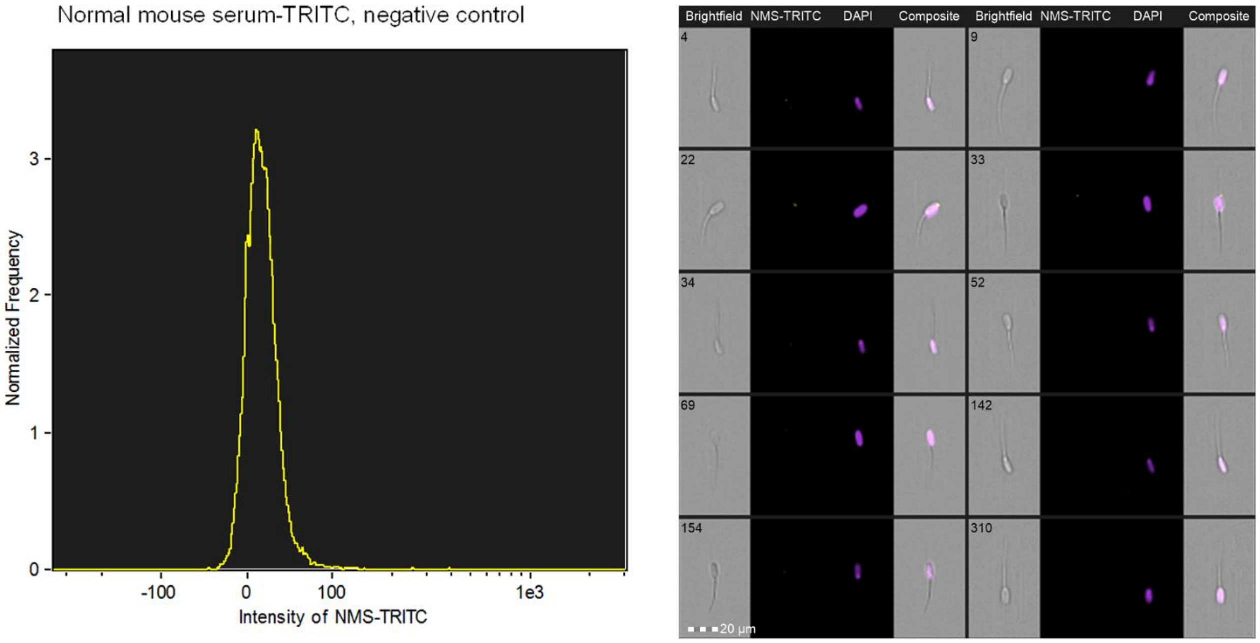

Supplementary Fig. 1: Flow cytometric sperm measurements combined with epifluorescence imaging of negative control. Non-immune, normal mouse serum, with comparable globulin concentration instead of primary antibody, and a corresponding secondary antibody conjugated with TRITC (1:150 dilution) were used. For fluorescence imaging, DAPI nuclear fluorescence dye was used as a contrast dye (1:1500 dilution). Flowcytometric run represents 10,000 events.

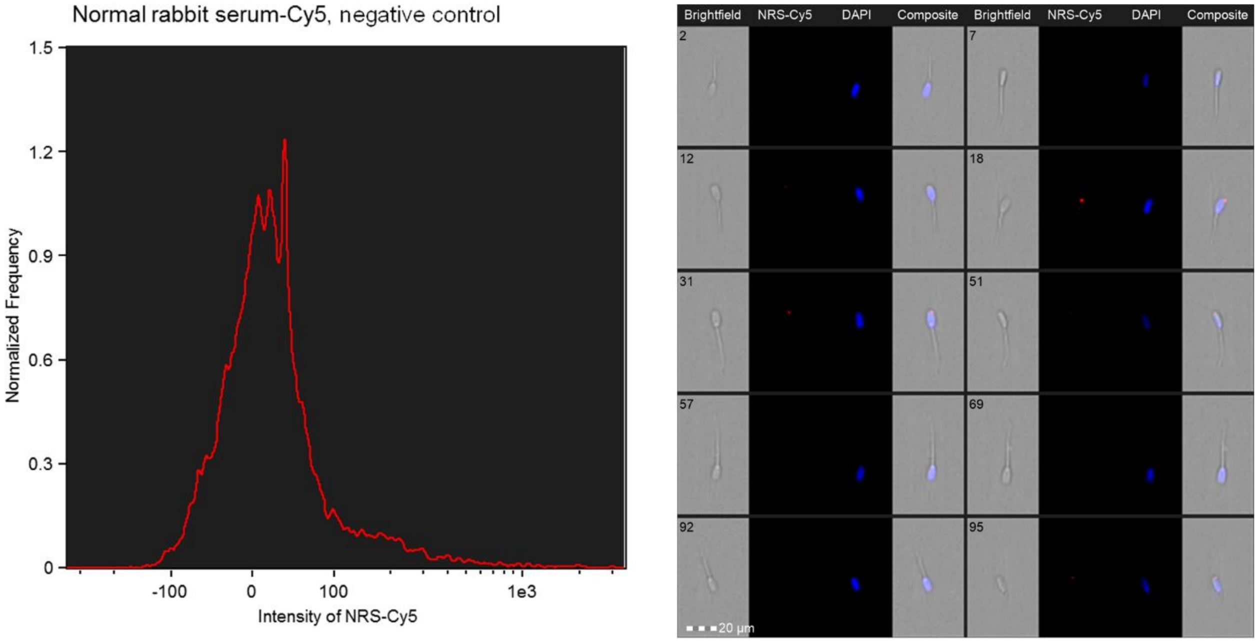

Supplementary Fig. 2: Flow cytometric sperm measurements combined with epifluorescence imaging of negative control. Non-immune normal rabbit serum, with comparable globulin concentration instead of primary antibody, and a corresponding secondary antibody conjugated with Cyanine 5 (1:150 dilution) were used. For fluorescence imaging, DAPI nuclear fluorescence dye was used as a contrast dye (1:1500 dilution). Flowcytometric run represents 10,000 events.

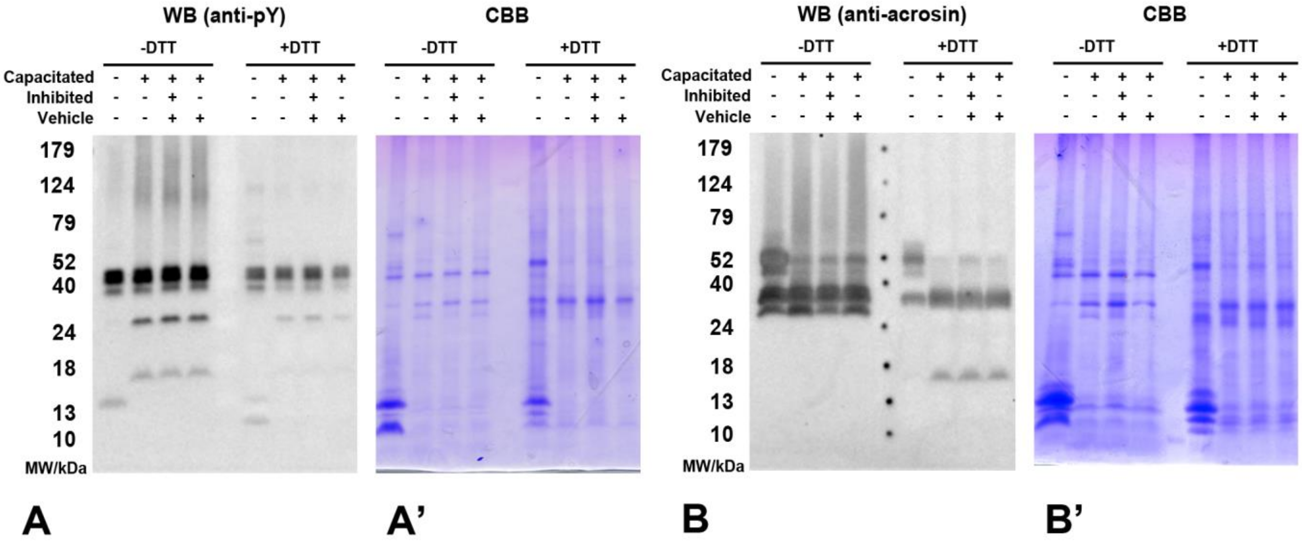

Supplementary Fig. 3: Protein load verification of Western blots shown in Fig. 1 B and C, including protein tyrosine phosphorylation (A), and proacrosin/acrosin conversion under non-reducing and reducing

conditions B). Respective residual gels (A'/B') shows roughly equal protein load per well after electrotransfer.

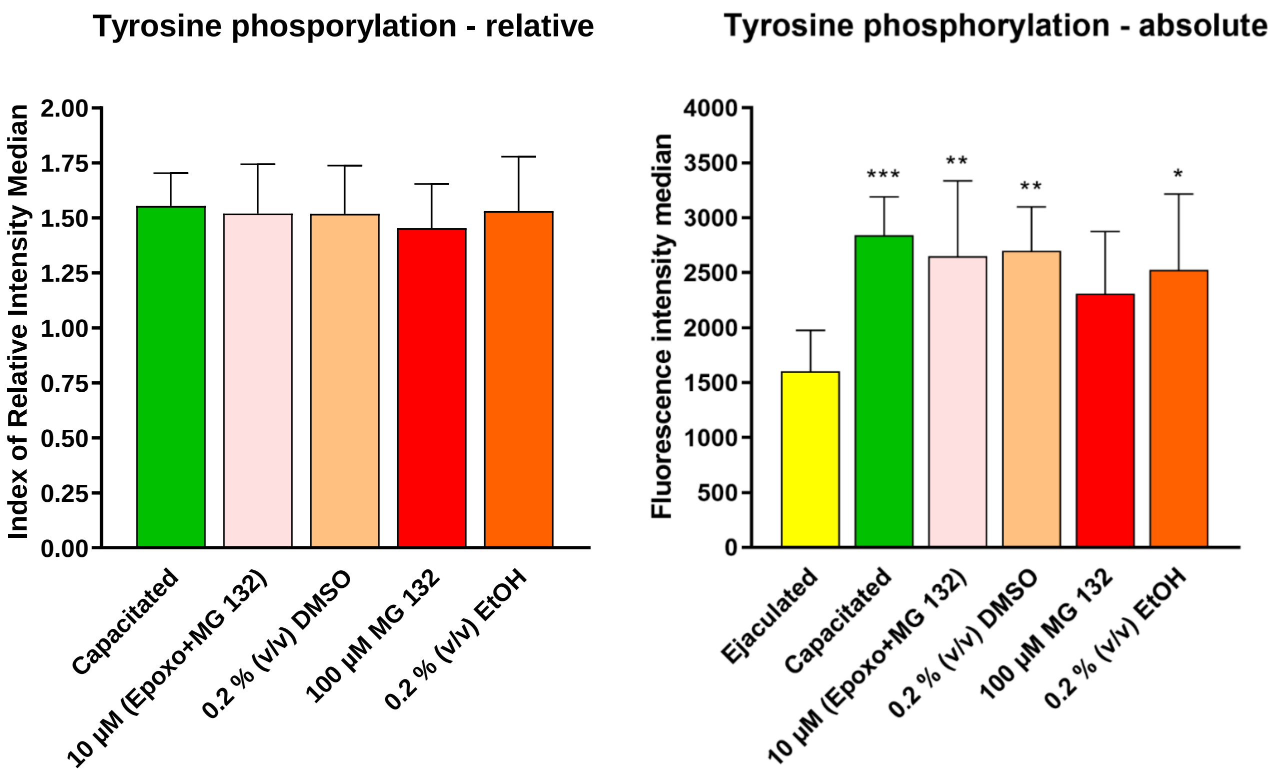

Supplementary Fig. 4: Bar graph representations of flow cytometric measurements of protein tyrosine phosphorylation from fig. 1A in relative and absolute representation. Results are presented as mean ± SD of four representative independent replicates. Statistical significance between different spermatozoa is denoted: *** for p-value ≤ 0.001, ** for 0.002 ≤ p-value < 0.033, and * for 0.033 ≤ p-value < 0.050.

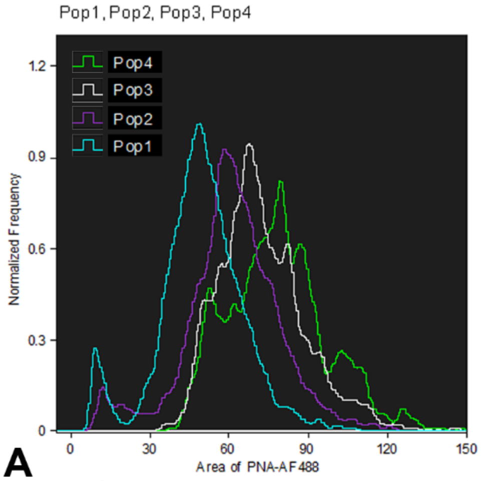

Sperm population	Statistical characteristics of populations: Count	Mean	Median	Mode
Population 1	3270	49.72	50.00	47.55
Population 2	2246	61.54	61.00	57.50
Population 3	1431	72.71	70.00	66.55
Population 4	731	78.97	78.00	80.12

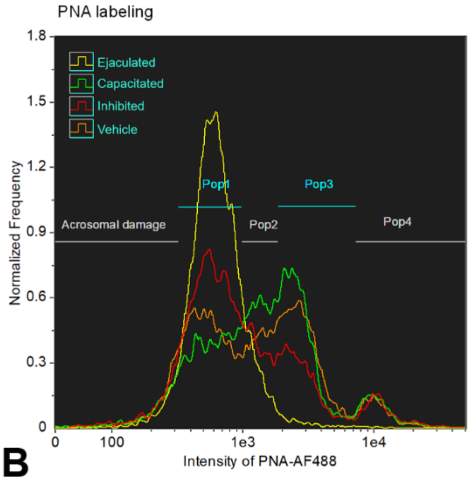

Sperm population (n=4)	Gated populations: Population 1	Population 2	Population 3	Population 4	Acros. damage
Ejaculated	85.99% ± 2.69%	9.95% ± 2.53%	1.22% ± 0.75%	0.12% ± 0.08%	2.14% ± 0.57%
Capacitated, no inhibition	22.59% ± 4.40%	22.48% ± 1.55%	40.96% ± 4.64%	6.93% ± 1.10%	6.74% ± 2.81%
Capacitated, 100 µM MG 132	45.78% ± 7.58%	21.10% ± 4.89%	20.50% ± 4.70%	4.99% ± 0.99%	7.07% ± 1.31%
Capacitated, 0.2 % EtOH	33.23% ± 3.24%	21.68% ± 2.72%	32.55% ± 2.35%	5.73% ± 0.83%	6.30% ± 1.97%

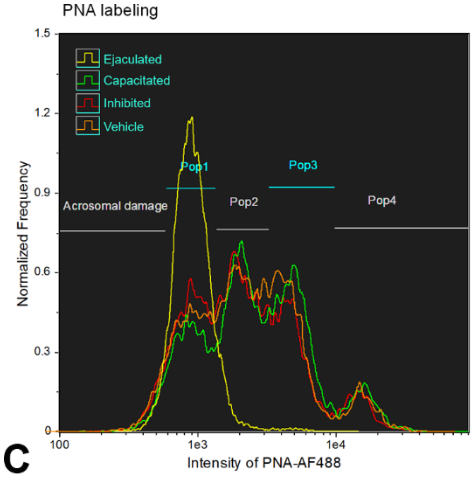

Sperm population (n=4)	Gated populations: Population 1	Population 2	Population 3	Population 4	Acros. damage
Ejaculated	87.04% ± 1.18%	8.73% ± 1.06%	0.81% ± 0.45%	0.16% ± 0.19%	3.11% ± 0.87%
Capacitated, no inhibition	22.36% ± 6.83%	33.08% ± 1.00%	30.60% ± 2.92%	7.38% ± 2.54%	7.79% ± 1.49%
Capacitated, 100 µM MG 132	28.44% ± 5.95%	34.68% ± 1.86%	23.73% ± 2.54%	6.39% ± 1.06%	7.97% ± 1.53%
Capacitated, 0.2 % EtOH	21.13% ± 5.19%	31.38% ± 3.95%	33.38% ± 3.95%	7.56% ± 1.80%	7.68% ± 0.71%

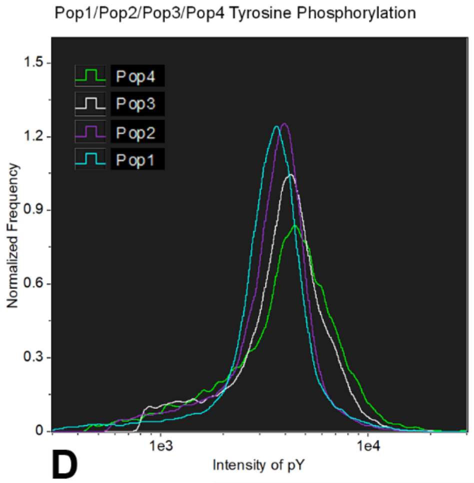

Sperm population	Statistical characteristics of populations: Count	Mean	Median	Mode
Population 1	24790	3633.91	3493.58	3336.44
Population 2	28287	3699.23	3672.68	3797.69
Population 3	29923	4222.26	4043.18	4080.75
Population 4	7690	4491.22	4207.21	4043.89

Supplementary Fig. 5: Flow cytometric measurements of the outer acrosomal membrane (OAM) remodeling during in vitro capacitation (IVC) under the proteasomal activity permissive/inhibiting conditions and with vehicle control. (A) Lectin PNA labeling area-quantification of individual populations from Fig. 2B. (B) Formaldehyde-fixed spermatozoa of fast-capacitating boar and (C) moderate capacitating boar were labeled with lectin PNA (peanut agglutinin). Same as in fresh, non-fixed spermatozoa (Fig. 2A), four sperm populations were distinguished and gated. Population gating and comparisons confirmed that at least populations 1, and 2 differ significantly ($P < 0.05$, marked red) when compared to vehicle control (0.2 % EtOH), depending on proteasomal inhibition (100 µM MG 132) during IVC. Unlike the experiment shown in Fig. 2, fresh, non-extended spermatozoa were used for all (ejaculated, capacitated with/without proteasomal inhibition, and vehicle control) groups. Results in (B and C) are presented as mean ± SD of four independent replicates. Every flow cytometric run represents

10,000 events. (D) Monitoring of protein tyrosine phosphorylation in four PNA populations as an approximation of the course of capacitation.

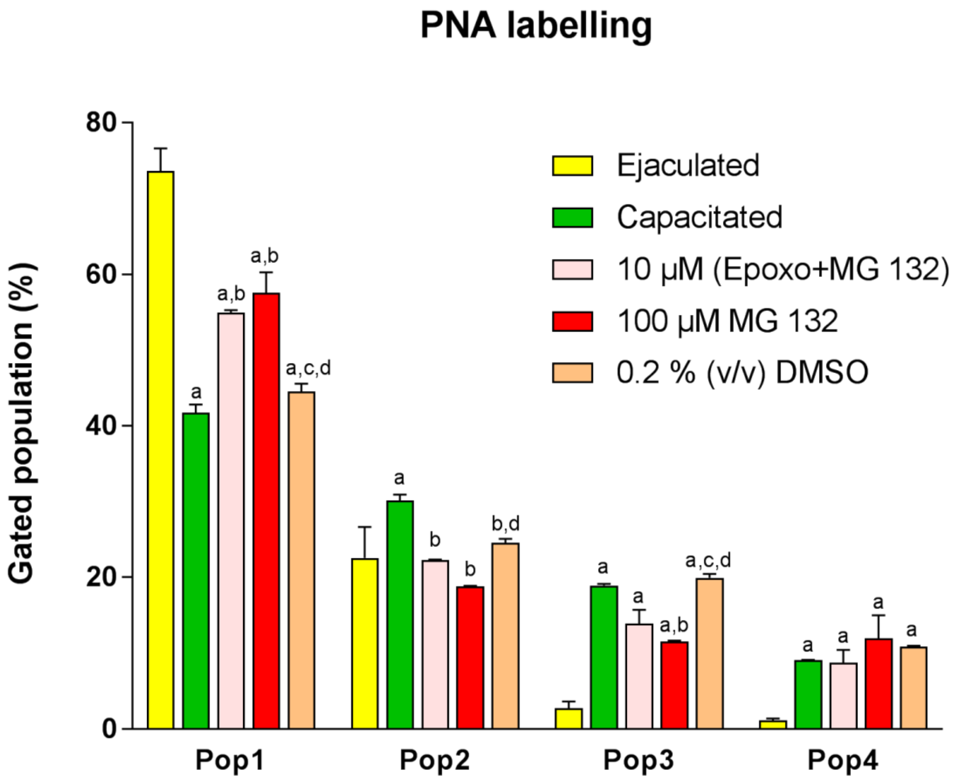

Supplementary Fig. 6: Bar graph representation of flow cytometric measurements of the outer acrosomal membrane (OAM) remodeling from fig. 2 during in vitro capacitation with proteasomal modulation and vehicle control. Results are presented as mean ± SD of four independent replicates. Sperm treatments are compared within each group (population) only. For simplicity, each bar within one population is statistically compared to all bars to its left only and the statistical significance with p-value < 0.05 is represented by a superscript of a corresponding bar.

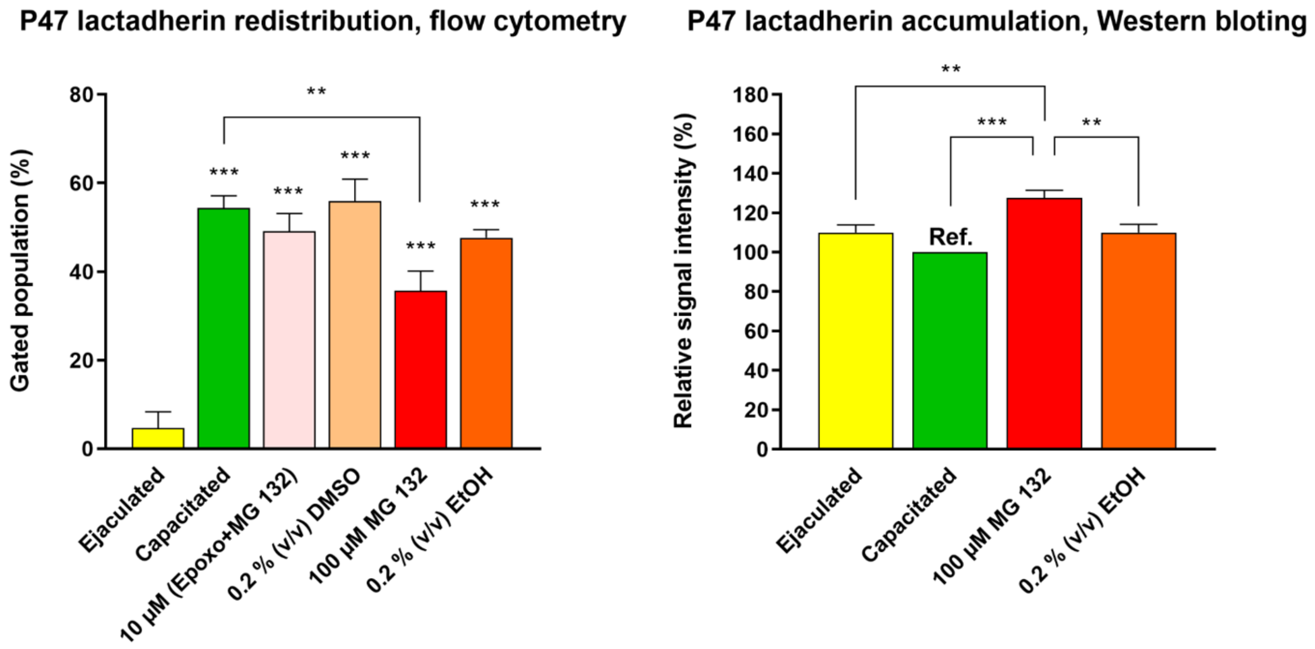

Supplementary Fig. 7: Bar graph representations of flow cytometric measurements of lactadherin MFGE8 redistribution from fig. 3A and Western blot detection of MFGE8 accumulation from fig. 3C. Results are presented as mean ± SD of four independent replicates. Statistical significance between different spermatozoa is denoted: *** for p-value ≤ 0.001, ** for 0.002 ≤ p-value < 0.033, and * for 0.033 ≤ p-value < 0.050.

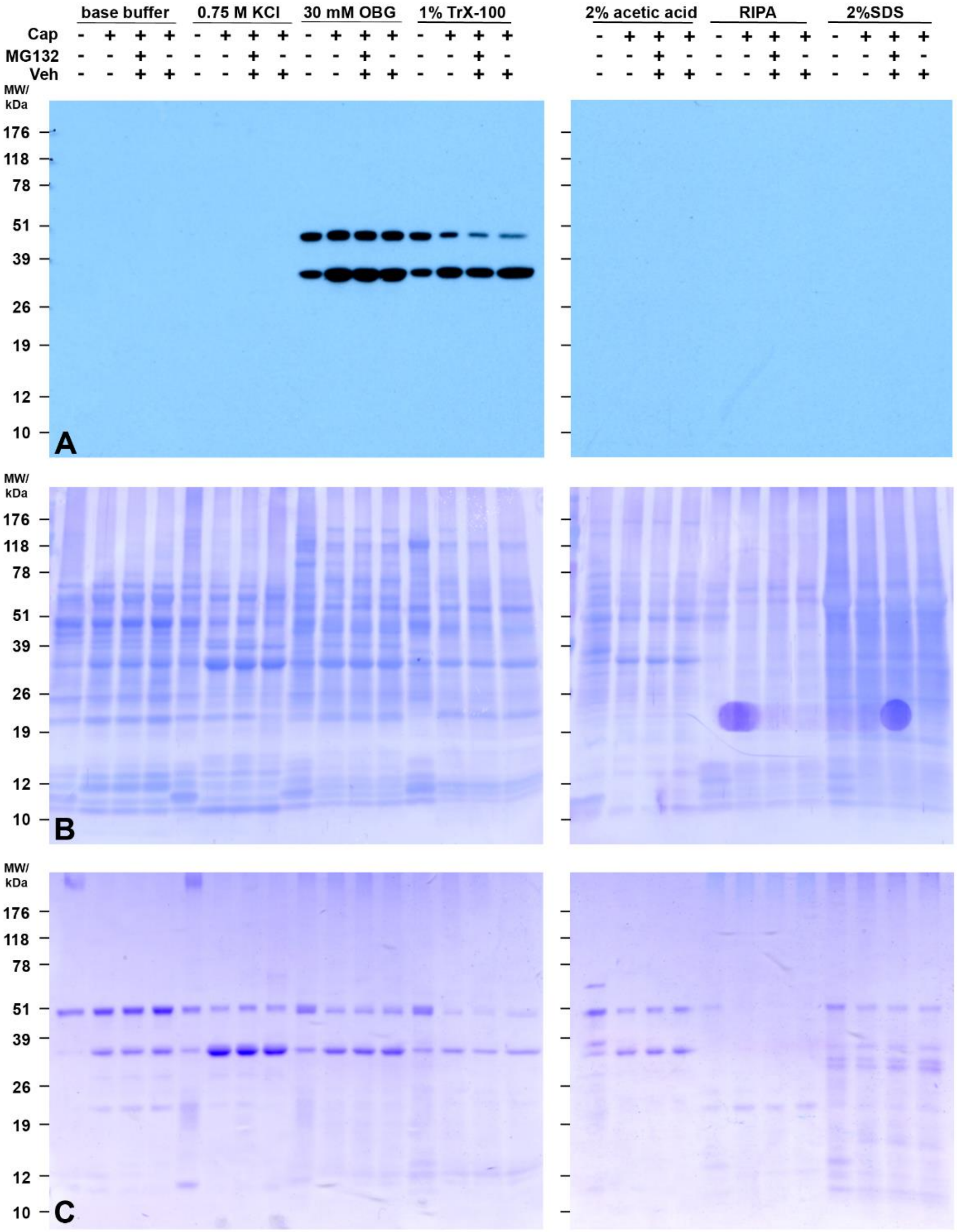

Supplementary Fig. 8: (A) Western blot detection of lactadherin MFGE8 in sequentially isolated proteins extracted from ejaculated and capacitated spermatozoa under proteasome permissive/inhibiting conditions (100 μM MG132) and vehicle control (0.2 % (v/v) EtOH). Lactadherin was completely extracted in the fourth extraction step (TrX-100), and no lactadherin signal was detected beyond this step. Pre-treatment of ejaculated spermatozoa in TBS and 0.75 M KCl made it possible to extract the 35 kDa form of MFGE8, as opposed to one-step extraction by TrX-100 in Fig. 3C. Blots were overexposed to include the weakest bands. (B) PVDF membrane stained with CBB after chemiluminescence detection shows comparable protein loads per lane within each treatment, (C) residual gel after electrotransfer for protein load

normalization purposes. Proteins were resolved on 4-20 % gradient gel under reducing conditions and protein equivalent of 20 million spermatozoa was loaded per single lane. The experiment was replicated five times with appropriate vehicle and loading controls.

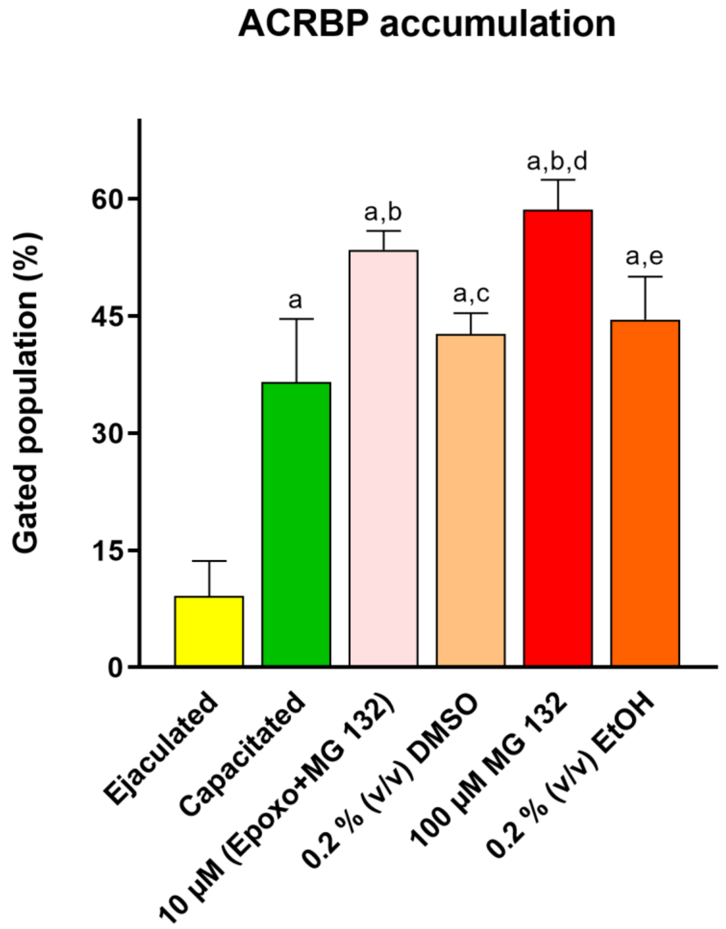

Supplementary Fig. 9: Bar graph representation of flow cytometric measurements of acrosin binding protein accumulation from fig. 4A. Results are presented as mean ± SD of four independent replicates. Statistical significance between different spermatozoa is denoted: *** for p-value ≤ 0.001, ** for 0.002 ≤ p-value < 0.033, and * for 0.033 ≤ p-value < 0.050.

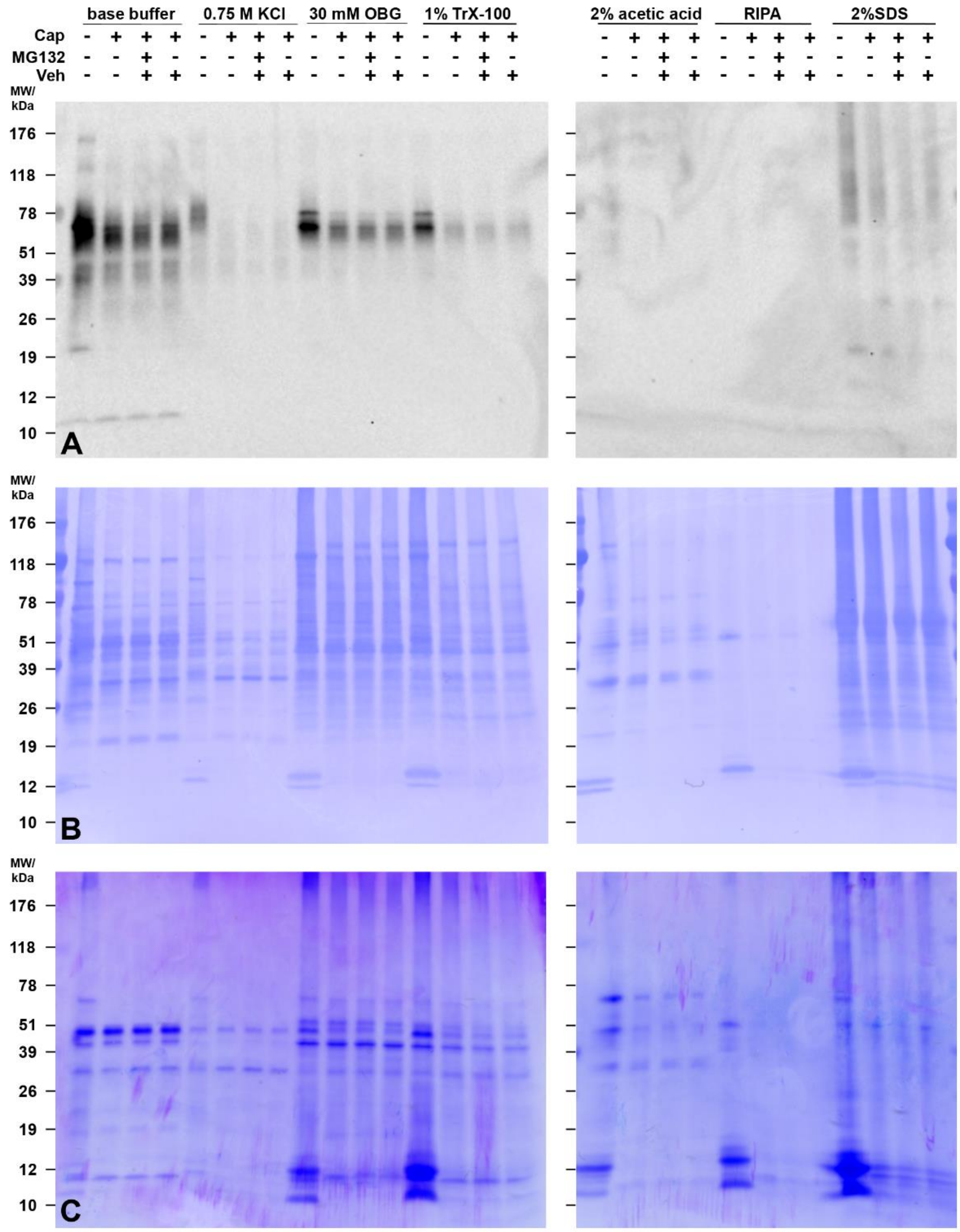

Supplementary Fig. 10: (A) Western blot detection of ACRBP (N-terminal part of the immature precursor protein and ACRBP degradation products were immunolabeled) in sequentially isolated proteins extracted from the ejaculated and capacitated spermatozoa under proteasome permissive/inhibiting conditions (100 μM MG132) including vehicle control (0.2 % (v/v) EtOH). ACRBP was completely extracted in the fourth extraction step (TrX-100) and no ACRBP signal was detected beyond this step. Including when the blots were overexposed, only a weak and non-specific binding of secondary antibody above 78 kDa was present in the last extraction step (2 % SDS). (B) PVDF membrane stained with CBB after chemiluminescence detection shows comparable protein loads per lane within each treatment, (C)

residual gel after electrotransfer for protein load normalization purposes. Proteins were resolved on 4-20 % gradient gel under reducing conditions and protein equivalent of 20 million spermatozoa was loaded per single lane. The experiment was replicated twice with comparable results.

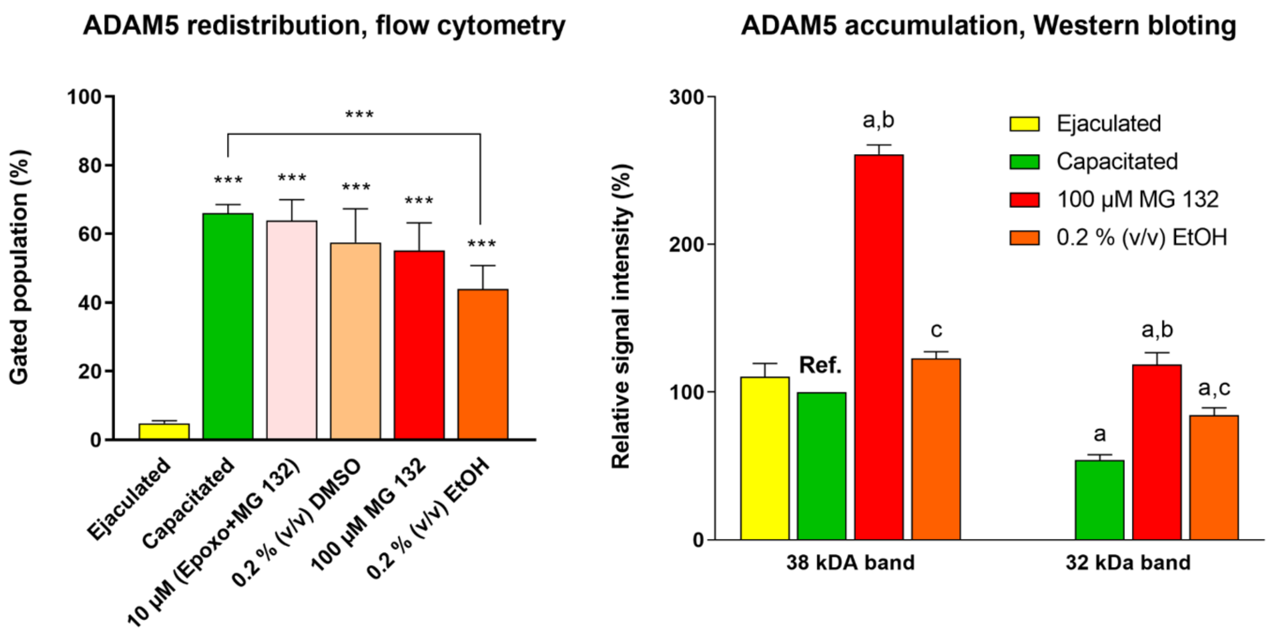

Supplementary Fig. 11: Bar graph representation of flow cytometric measurements of ADAM5 redistribution from fig. 5A and grouped bar graph representation of Western blot detection of ADAM5 accumulation from fig. 5C. Results are presented as mean ± SD of four independent replicates for flow cytometry, and three independent replicates for Western blotting. For ADAM5 redistribution, statistical significance between different spermatozoa is denoted: *** for p-value < 0.001, ** for 0.002 ≤ p-value < 0.033, and * for 0.033 ≤ p-value < 0.050. For ADAM5 accumulation, sperm treatments are compared for each protein band individually. For simplicity, each bar within one group (protein band) is compared to all the bars to its left only, and the statistical significance with a p-value < 0.050 is represented by a small letter of the corresponding bar.

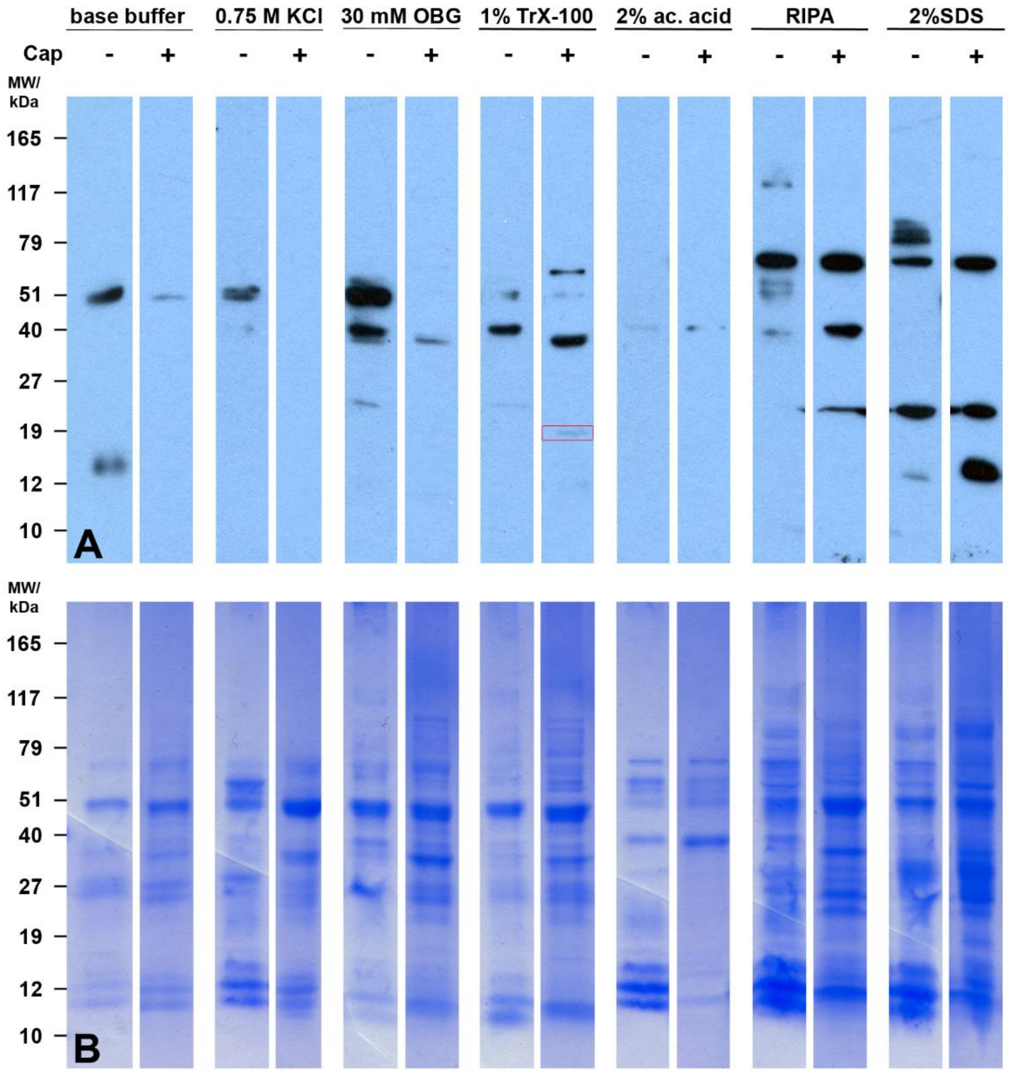

Supplementary Fig. 12: (A) Western blot detection of ADAM5 in sequentially isolated proteins extracted from the ejaculated and capacitated spermatozoa. Different processed forms with various solubility of ADAM5 are detected within each extraction step. It is apparent that the accumulation of 38 kDa band in Fig. 5 is caused by a change in solubility, as the 38 kDa band is detected in the sixth extraction (RIPA) step in capacitated vs. ejaculated spermatozoa. The absence of 32 kDa band and the presence of 19 kDa band (red rectangle) in capacitated spermatozoa may suggest differential ADAM5 processing during capacitation between two individual boars. Blots were overexposed to expose the weakest bands. (B) Residual gel after electrotransfer for protein load normalization purposes. Proteins were resolved on 4-20 % gradient gel under reducing conditions, protein equivalent of 20 million ejaculated or 30 million capacitated spermatozoa was loaded per single lane. The experiment was replicated twice with comparable results. Ejaculated samples were run separately from capacitated, and the blots were spliced and re-arranged for better comparison within each isolation step between ejaculated and capacitated spermatozoa.

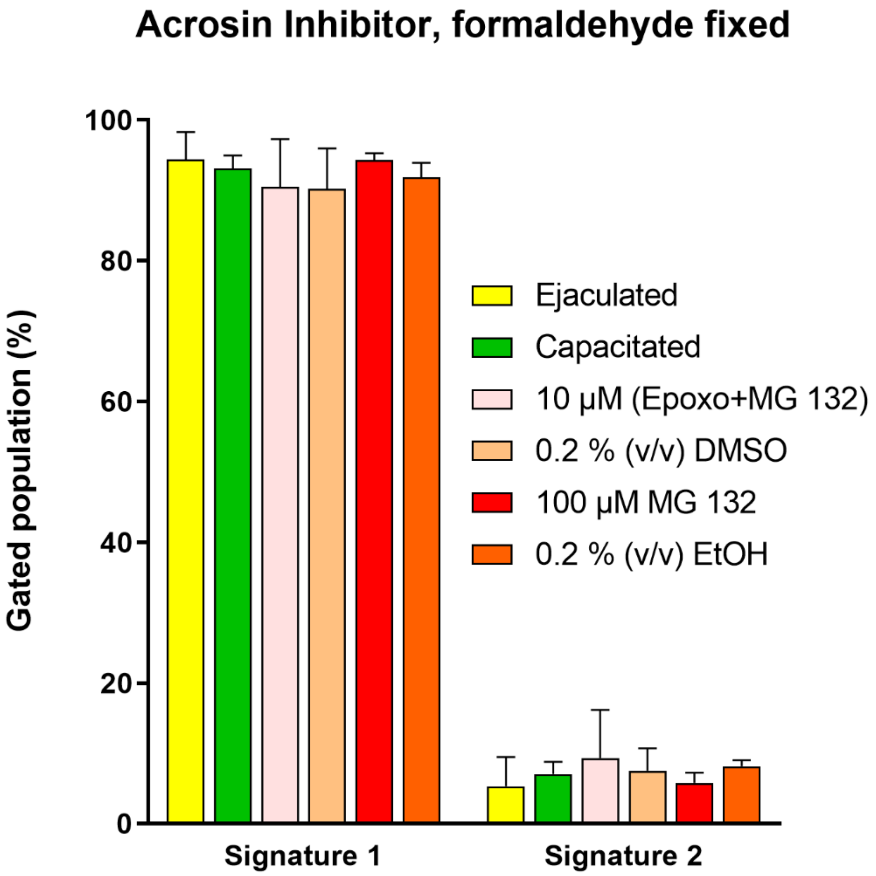

Supplementary Fig. 13: Bar graph representations of flow cytometric measurements of sperm associated acrosin inhibitor (SAAI) processing during in vitro capacitation with proteasomal modulation and vehicle control from Fig. 6. Two distinctly labeled populations, termed *Signature 1* and *2* have been observed. Results are presented as mean ± SD of four independent replicates. Sperm treatments are compared within each group (population) only. For simplicity, each bar within one population is compared to all the bars to its left only and the statistical significance with a p-value < 0.050 is represented by a small letter of the corresponding bar. No statistical significance was observed.

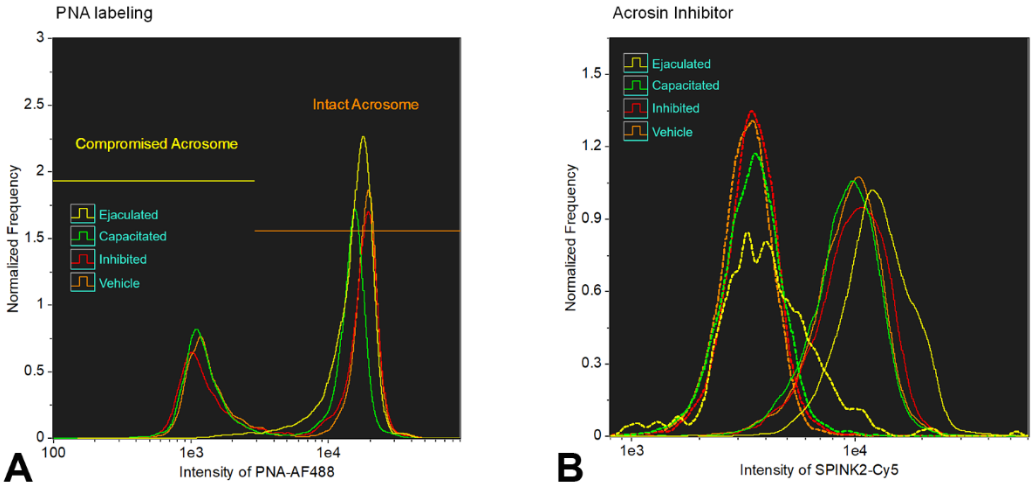

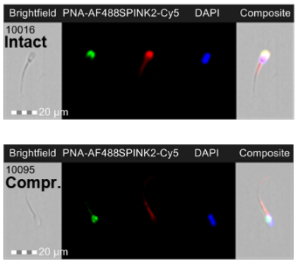

Sperm population (n=4)		PNA labeling: Acrosome intact spermatozoa	Relative Intensity Median Index: Acrosome intact spermatozoa	Relative Intensity Median Index: Acrosome compromised spermatozoa
Ejaculated		95.70% ± 1.11%	Taken as the base of Indexes	
Capacitated, no inhibition		76.75% ± 2.87%	0.72 ± 0.03	0.85 ± 0.16
Capacitated,	10 µM (Epoxo+MG 132)	71.86% ± 7.95%	0.78 ± 0.08	0.81 ± 0.17
	0.2 % DMSO	70.39% ± 10.67%	0.72 ± 0.06	0.84 ± 0.20
Capacitated,	100 µM MG 132	74.83% ± 4.66%	0.74 ± 0.03	0.87 ± 0.21
	0.2 % EtOH	66.31% ± 15.09%	0.70 ± 0.07	0.82 ± 0.21

Supplementary Fig. 14: (A) Flow cytometric measurements of outer acrosomal membrane (OAM) labeling with PNA lectin after acetone fixation in ejaculated, and in vitro capacitated spermatozoa under proteasome permissive/inhibiting conditions (10 µM epoxomicin + 10 µM MG 132 for mild inhibiting conditions, and 100 µM MG132 for strong inhibiting conditions) and vehicle control, combined with epifluorescence imaging of PNA lectin labeling and SPINK2 localization in spermatozoa with intact and compromised acrosome. (B) Flow cytometric measurements of SPINK2 intensity of spermatozoa with intact (solid line) vs compromised (dashed line) acrosomes in ejaculated, and in vitro capacitated spermatozoa under proteasome permissive/inhibiting conditions (10 µM epoxomicin + 10 µM MG 132 for mild inhibiting conditions, and 100 µM MG132 for strong inhibiting conditions) and vehicle control. Every flow cytometric run represents 10,000 events. Results are presented as mean ± SD of four independent replicates. No statistical significance was observed between spermatozoa capacitated under proteasomal inhibition and its corresponding vehicle control.

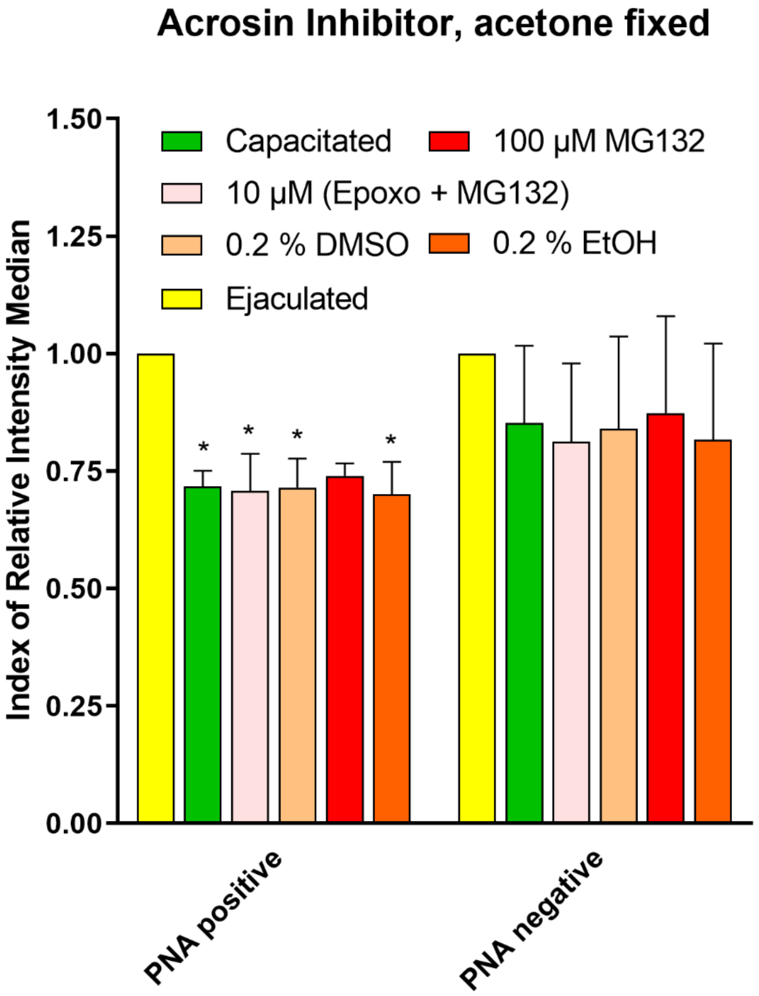

Supplementary Fig. 15: Bar graph representations of flow cytometric measurements of sperm associated acrosin inhibitor (SAAI) processing during in vitro capacitation with proteasomal modulation and vehicle control from Fig. S12 table. Significant decrease in fluorescence intensity median was observed in acrosome intact spermatozoa of all treatments ($P<0.050$) except 100 µM MG132 ($P=0.139$), and acrosome damaged spermatozoa ($0.462<P<0.959$). Proteasomal inhibition had no significant impact on SPINK2 accumulation during IVC ($P>0.999$). Results are presented as mean ± SD of four independent replicates. Sperm treatments are compared within each group (population) only. For simplicity, each bar within one population is compared to all the bars to its left only and the statistical significance with p-value < 0.050 is represented by an asterisk.

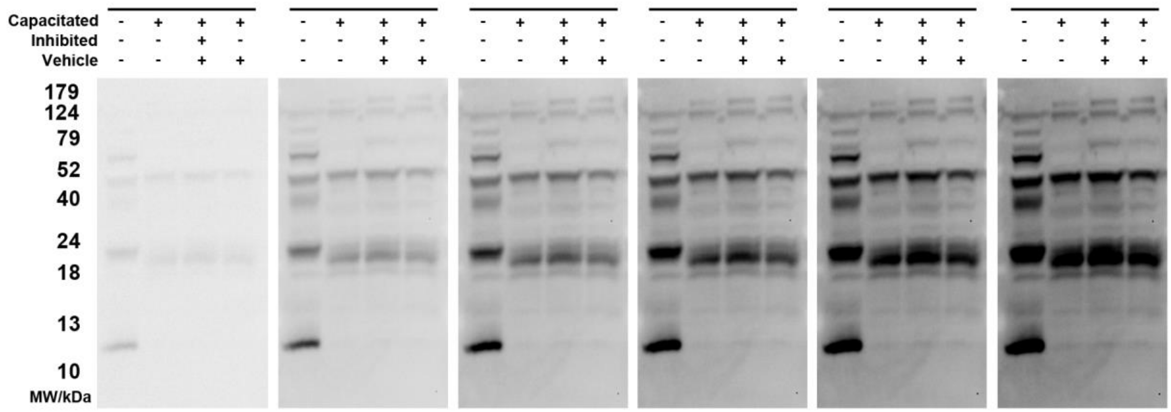

Supplementary Fig. 16: Full-length blot from Fig 6C at different exposure times.

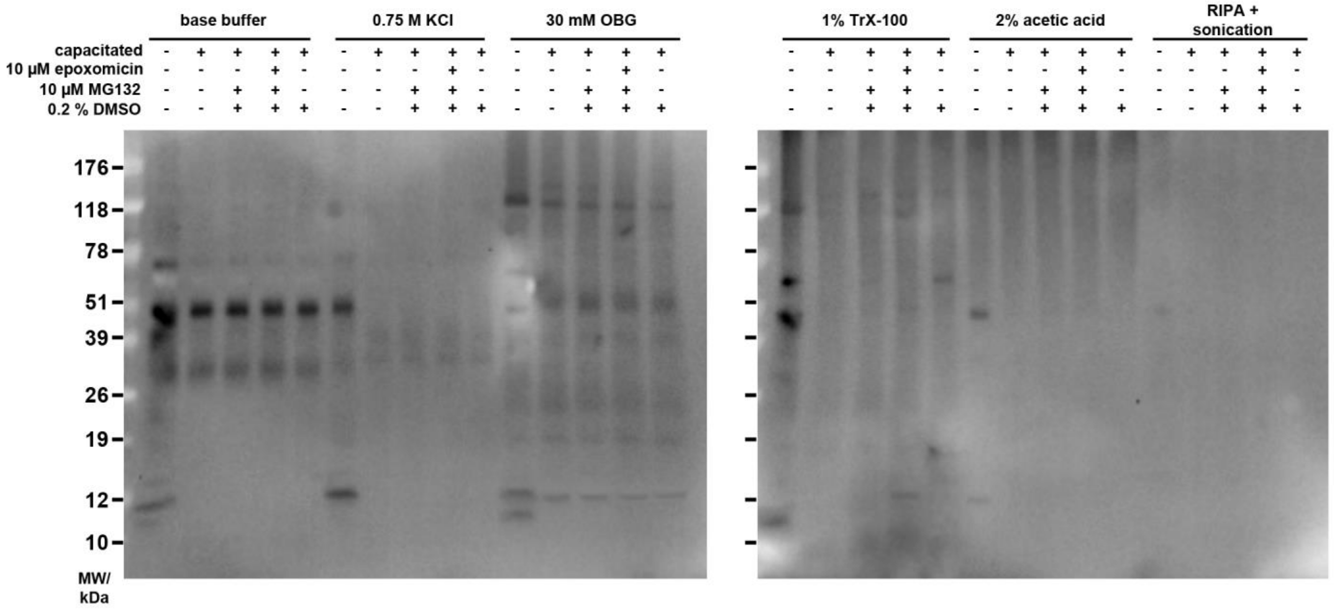

Supplementary Fig. 17: Full-length blot from Fig 6D.

Description and proposed function of MS/MS identified proteins from Tab.1

We identified two differentially accumulated protein bands (#1, 3) by parallel isolation approach and four protein bands (#1-4) by sequential extraction. Bands (#5, 5') were found to be acrosin. From the mass spectrometric results (Tab.1), both bands #1 and #2 contained lactadherin, MFGE8, a protein proposed to have multiple potential roles in the reproductive process such as an integrin RGD-dependent ligand [1,2], a zona pellucida (ZP) receptor [3-5], an anchor or interactor for the sperm 26 proteasome [6], a signaling cascade primer/element during sperm capacitation [7] or an oviductal epithelia cell/sperm reservoir receptor [8]. Furthermore, both bands #1, 2 contained acrosin, a major component of acrosomal content that has been shown to have a role in reproduction as a secondary ZP-binding receptor [9]. Although the acrosin knock

out mice are fertile [10], in animals with a thicker ZP such as pigs (16-20 μm) acrosin may be essential for sperm-zona penetration [11]. Significant peptide matches exclusive to band #1 was cathepsin F, papain-like cysteine proteases reported in prostasomes [12] and seminal plasma [13]; however, those matching peptides were of a lower relative abundance compared to MFGE8 and ACR. Another major protein in band #2 was zona-pellucida binding protein 2 (ZPBP2), which has intra-acrosomal localization in the mouse; male mice null for ZPBP2 displayed subfertility associated with the defects in ZP interactions [14]. Recently, ZPBP2 was found to be associated with t-complex 1/chaperonin-containing multimeric protein (TCP1) complexes, isolated from the sperm plasma membrane [15,16]. Thus, the capacitation dependent relocation of ZPBP2 from the acrosome to the sperm surface could follow the redistribution of sperm proteins ZP3R/sp56, ZAN, proacrosin/acrosin, ACRBP/sp32, ASPX/SP-10 and ZPBP1, thought to translocate to the sperm surface during capacitation [17,18]. Our results support the participation of UPS in ZPBP2 and/or acrosin relocalization as the band #2 isolated by 0.75 M KCl, presumably from sperm surface [19], was less abundant in spermatozoa capacitated with proteasomal inhibition. Last abundant protein predicted in band #2 was malate dehydrogenase 1 (MDH), a key protein in cellular respiration with two distinguished isoforms, a cytosolic and a mitochondrial one; also implicated in boar fertility as candidate littler size predictors [20].

Since the molar masses of bands #3 and 4 differed only by 2 kDa and the extraction reagent was TrX-100 for both, it is not surprising that some relevant peptides for each protein were present in both bands. This was the case of tubulin beta 4B class IVb (TUBB4B), a major cytoskeletal component of microtubules found in the sperm tail axoneme and together with dynein. Another predicted protein shared by bands #3 and 4 was leucyl and cystinyl aminopeptidase/cytosol aminopeptidase (LNPEP) belonging to a group of exopeptidase metalloenzymes containing Zn^{2+} ion and catalyzing the cleavage of an amino-terminal residue of substrate proteins. The presence of aminopeptidase has been described in mammalian and sea urchin spermatozoa [21-26]. The interaction between sperm aminopeptidase and molecules on the oocyte surface may play a role in the induction of acrosomal exocytosis in a mussel M. edulis [27]. Functional studies of aminopeptidase in different species have not been reported yet. The ATP synthase, H+ transporting, mitochondrial F1 complex, alpha subunit 1, cardiac muscle (ATP5A1) has been also identified in both #3 and 4 bands. Rat spermatozoa acquire ATP5A1 during the epididymal passage; it was identified in the sperm head extract [28], implying a non-canonical localization/function in spermatozoa. Identified exclusively in band #3, dihydrolipoamide dehydrogenase (DLD), a post-pyruvate metabolic enzyme is typically localized in mitochondria in the eukaryotic systems [29]. However, non-canonical extra-mitochondrial localization of this enzyme has been reported in mammalian spermatozoa, confined to the acrosome and sperm tail principal piece [30], and the enzyme seems to have bi-directional activity. DLD undergoes capacitation-dependent phosphorylation, with a different time course for acrosomal and principal piece localized enzyme, which is required for hyperactivation and acrosome reaction of hamster spermatozoa [31]. The 26S proteasome may, therefore, regulate the activity of this enzyme via protein phosphorylation loop as suggested by Morales et al. [32]. CD46 molecule pseudogene 1/membrane cofactor protein/MCP/CD46-like (CD46P1) was another protein predicted in band #3. The CD46 is known as a complement receptor protecting cells from complement attack in humans and is localized on the inner acrosomal membrane of human and rodent spermatozoa [33]. Proposed functions include the protection of

acrosome-reacted spermatozoa from complement attack in the female reproductive tract [34,35] and the gamete fusion protein in hamster [36] and rat spermatozoa [37]. However, the CD46 KO mouse retained fertility, while they are prone to spontaneous acrosomal exocytosis, suggesting that CD46 may be involved in the regulation of acrosomal function [38].

Aldehyde dehydrogenase 2 family (ALDH2) was exclusive to band #4, a detoxifying enzyme responsible for the catalytic conversion of acetaldehyde into acetate [39]. This enzyme plays an important role in the reproductive toxicity of ALDH2 substrates. Treatment of ALDH2 KO mice with ethylene glycol monoethyl ether and ethyl tertiary butyl ether (both are substrates or ALDH2) resulted in decreased sperm motility [40,41]. Aldehyde dehydrogenases 1A3, 1B1, and 2 play a pivotal role in the maintenance of stallion sperm motility [42]. ALDH2 was reported to be localized non-canonically, on the entire stallion sperm with the strongest signal on the head and tail midpiece [42], as well as identified in rat sperm head extract [28].

Aldehyde dehydrogenase 7 family member A1/alpha-aminoadipic semialdehyde dehydrogenase/antiquitin (ALDH7A1) was also predicted in band #4. Similar to ALDH2, ALDH7A1 has a protective function specifically against osmotic and oxidative stress caused by lipid peroxidation-derived aldehydes [43]. The ALDH7A1 has not yet been reported in spermatozoa, though it has been previously reported in mice testes; the protein was found to be localized in the cytosol, mitochondria, and nucleus of somatic cells [43]. Altogether, the proteins identified or predicted in bands #1-4 may be regulated by UPS during sperm capacitation. Individual study of proteins from Table 1 not yet shown to be regulated by UPS is the next logical step in the investigation of the UPS role in sperm capacitation.

References

1 Ensslin, M. *et al.* Molecular cloning and characterization of P47, a novel boar sperm-associated zona pellucida-binding protein homologous to a family of mammalian secretory proteins. *Biology of reproduction* **58**, 1057-1064 (1998).

2 Andersen, M. H., Berglund, L., Rasmussen, J. T. & Petersen, T. E. Bovine PAS-6/7 binds alpha v beta 5 integrins and anionic phospholipids through two domains. *Biochemistry* **36**, 5441-5446, doi:10.1021/bi963119m (1997).

3 Ensslin, M. A. & Shur, B. D. Identification of mouse sperm SED1, a bimotif EGF repeat and discoidin-domain protein involved in sperm-egg binding. *Cell* **114**, 405-417 (2003).

4 Petrunkina, A. M., Lakamp, A., Gentzel, M., Ekhlasi-Hundrieser, M. & Topfer-Petersen, E. Fate of lactadherin P47 during post-testicular maturation and capacitation of boar spermatozoa. *Reproduction (Cambridge, England)* **125**, 377-387 (2003).

5 Zigo, M. *et al.* Panel of monoclonal antibodies to sperm surface proteins as a tool for monitoring localization and identification of sperm-zona pellucida receptors. *Cell Tissue Res* **359**, 895-908, doi:10.1007/s00441-014-2072-9 (2015).

6 Miles, E. L. *et al.* Transgenic pig carrying green fluorescent proteasomes. *Proceedings of the National Academy of Sciences of the United States of America* **110**, 6334-6339, doi:10.1073/pnas.1220910110 (2013).

7 Nagdas, S. K., Smith, L., Medina-Ortiz, I., Hernandez-Encarnacion, L. & Raychoudhury, S. Identification of bovine sperm acrosomal proteins that interact with a 32-kDa acrosomal matrix protein. *Molecular and cellular biochemistry* **414**, 153-169, doi:10.1007/s11010-016-2668-3 (2016).

8 Silva, E., Frost, D., Li, L., Bovin, N. & Miller, D. J. Lactadherin is a candidate oviduct Lewis X trisaccharide receptor on porcine spermatozoa. *Andrology* **5**, 589-597, doi:10.1111/andr.12340 (2017).

9 Jones, R. & Williams, R. M. Identification of zona- and fucoidan-binding proteins in guinea-pig spermatozoa and mechanism of recognition. *Development (Cambridge, England)* **109**, 41-50 (1990).

10 Baba, T., Azuma, S., Kashiwabara, S. & Toyoda, Y. Sperm from mice carrying a targeted mutation of the acrosin gene can penetrate the oocyte zona pellucida and effect fertilization. *The Journal of biological chemistry* **269**, 31845-31849 (1994).

11 Topfer-Petersen, E., Ekhlasi-Hundrieser, M. & Tsolova, M. Glycobiology of fertilization in the pig. *The International journal of developmental biology* **52**, 717-736, doi:10.1387/ijdb.072536et (2008).

12 Utleg, A. G. *et al.* Proteomic analysis of human prostasomes. *The Prostate* **56**, 150-161, doi:10.1002/pros.10255 (2003).

13 Pilch, B. & Mann, M. Large-scale and high-confidence proteomic analysis of human seminal plasma. *Genome biology* **7**, R40, doi:10.1186/gb-2006-7-5-r40 (2006).

14 Lin, Y. N., Roy, A., Yan, W., Burns, K. H. & Matzuk, M. M. Loss of zona pellucida binding proteins in the acrosomal matrix disrupts acrosome biogenesis and sperm morphogenesis. *Molecular and cellular biology* **27**, 6794-6805, doi:10.1128/mcb.01029-07 (2007).

15 Redgrove, K. A. *et al.* Involvement of multimeric protein complexes in mediating the capacitation-dependent binding of human spermatozoa to homologous zonae pellucidae. *Developmental biology* **356**, 460-474, doi:10.1016/j.ydbio.2011.05.674 (2011).

16 Dun, M. D. *et al.* The chaperonin containing TCP1 complex (CCT/TRiC) is involved in mediating sperm-oocyte interaction. *The Journal of biological chemistry* **286**, 36875-36887, doi:10.1074/jbc.M110.188888 (2011).

17 Wassarman, P. M. Mammalian fertilization: the strange case of sperm protein 56. *BioEssays : news and reviews in molecular, cellular and developmental biology* **31**, 153-158, doi:10.1002/bies.200800152 (2009).

18 Kongmanas, K. *et al.* Proteomic Characterization of Pig Sperm Anterior Head Plasma Membrane Reveals Roles of Acrosomal Proteins in ZP3 Binding. *Journal of cellular physiology* **230**, 449-463, doi:10.1002/jcp.24728 (2015).

19 Belleannee, C. *et al.* Purification and identification of sperm surface proteins and changes during epididymal maturation. *Proteomics* **11**, 1952-1964, doi:10.1002/pmic.201000662 (2011).

20 Kwon, W. S. *et al.* Discovery of predictive biomarkers for litter size in boar spermatozoa. *Molecular & cellular proteomics : MCP* **14**, 1230-1240, doi:10.1074/mcp.M114.045369 (2015).

21 Arienti, G., Carlini, E., Verdacchi, R., Cosmi, E. V. & Palmerini, C. A. Prostasome to sperm transfer of CD13/aminopeptidase N (EC 3.4.11.2). *Biochimica et biophysica acta* **1336**, 533-538 (1997).

22 Arienti, G., Carlini, E., Verdacchi, R. & Palmerini, C. A. Transfer of aminopeptidase activity from prostasomes to sperm. *Biochimica et biophysica acta* **1336**, 269-274 (1997).

23 Hansbrough, J. R. & Garbers, D. L. Speract. Purification and characterization of a peptide associated with eggs that activates spermatozoa. *The Journal of biological chemistry* **256**, 1447-1452 (1981).

24 Schaller, J. & Glander, H. J. Flow cytometric analysis of enzymes in live spermatozoa before and after cryostorage. *Andrologia* **32**, 357-364 (2000).

25 Yasuhara, T., Yokosawa, H., Hoshi, M. & Ishii, S. Sea urchin sperm aminopeptidase: comparative studies of sperm-associated and -solubilized enzymes. *Biochemistry international* **7**, 593-598 (1983).

26 Yasuhara, T., Yokosawa, H. & Ishii, S. Purification and characterization of an aminopeptidase from sperm of the sea urchin, Strongylocentrotus intermedius. Ca2(+)-dependent substrate specificity as a novel feature of the enzyme. *Journal of biochemistry* **107**, 273-279 (1990).

27 Togo, T. & Morisawa, M. GPI-anchored aminopeptidase is involved in the acrosome reaction in sperm of the mussel mytilusedulis. *Molecular reproduction and development* **67**, 465-471, doi:10.1002/mrd.20037 (2004).

28 Suryawanshi, A. R., Khan, S. A., Gajbhiye, R. K., Gurav, M. Y. & Khole, V. V. Differential proteomics leads to identification of domain-specific epididymal sperm proteins. *Journal of andrology* **32**, 240-259, doi:10.2164/jandrol.110.010967 (2011).

29 Patel, M. S. & Roche, T. E. Molecular biology and biochemistry of pyruvate dehydrogenase complexes. *FASEB journal : official publication of the Federation of American Societies for Experimental Biology* **4**, 3224-3233 (1990).

30 Mitra, K., Rangaraj, N. & Shivaji, S. Novelty of the pyruvate metabolic enzyme dihydrolipoamide dehydrogenase in spermatozoa: correlation of its localization, tyrosine phosphorylation, and activity during sperm capacitation. *The Journal of biological chemistry* **280**, 25743-25753, doi:10.1074/jbc.M500310200 (2005).

31 Mitra, K. & Shivaji, S. Novel tyrosine-phosphorylated post-pyruvate metabolic enzyme, dihydrolipoamide dehydrogenase, involved in capacitation of hamster spermatozoa. *Biology of reproduction* **70**, 887-899, doi:10.1095/biolreprod.103.022780 (2004).

32 Morales, P., Diaz, E. S. & Kong, M. Proteasome activity and its relationship with protein phosphorylation during capacitation and acrosome reaction in human spermatozoa. *Society of Reproduction and Fertility supplement* **65**, 269-273 (2007).

33 Seya, T. *et al.* CD46 (membrane cofactor protein of complement, measles virus receptor): structural and functional divergence among species (review). *International journal of molecular medicine* **1**, 809-816 (1998).

34 Cervoni, F. *et al.* Identification and characterization of membrane cofactor protein of human spermatozoa. *Journal of immunology (Baltimore, Md. : 1950)* **148**, 1431-1437 (1992).

35 Seya, T. *et al.* Membrane cofactor protein (MCP, CD46) in seminal plasma and on spermatozoa in normal and "sterile" subjects. *European journal of immunology* **23**, 1322-1327, doi:10.1002/eji.1830230620 (1993).

36 Okabe, M. *et al.* A human sperm antigen possibly involved in binding and/or fusion with zona-free hamster eggs. *Fertility and sterility* **54**, 1121-1126 (1990).

37 Mizuno, M., Harris, C. L., Johnson, P. M. & Morgan, B. P. Rat membrane cofactor protein (MCP; CD46) is expressed only in the acrosome of developing and mature spermatozoa and mediates binding to immobilized activated C3. *Biology of reproduction* **71**, 1374-1383, doi:10.1095/biolreprod.104.030114 (2004).

38 Inoue, N. *et al.* Disruption of mouse CD46 causes an accelerated spontaneous acrosome reaction in sperm. *Molecular and cellular biology* **23**, 2614-2622 (2003).

39 Ehrig, T., Bosron, W. F. & Li, T. K. Alcohol and aldehyde dehydrogenase. *Alcohol and alcoholism (Oxford, Oxfordshire)* **25**, 105-116 (1990).

40 Wang, R. S. *et al.* Reproductive toxicity of ethylene glycol monoethyl ether in Aldh2 knockout mice. *Industrial health* **45**, 574-578 (2007).

41 Weng, Z. *et al.* Assessment of the reproductive toxicity of inhalation exposure to ethyl tertiary butyl ether in male mice with normal, low active and inactive ALDH2. *Archives of toxicology* **88**, 1007-1021, doi:10.1007/s00204-014-1192-z (2014).

42 Gibb, Z., Lambourne, S. R., Curry, B. J., Hall, S. E. & Aitken, R. J. Aldehyde Dehydrogenase Plays a Pivotal Role in the Maintenance of Stallion Sperm Motility. *Biology of reproduction* **94**, 133, doi:10.1095/biolreprod.116.140509 (2016).

43 Brocker, C. *et al.* Aldehyde dehydrogenase 7A1 (ALDH7A1) is a novel enzyme involved in cellular defense against hyperosmotic stress. *The Journal of biological chemistry* **285**, 18452-18463, doi:10.1074/jbc.M109.077925 (2010).
